# Validation and Genotyping of Multiple Human Polymorphic Inversions Mediated by Inverted Repeats Reveals a High Degree of Recurrence

**DOI:** 10.1371/journal.pgen.1004208

**Published:** 2014-03-20

**Authors:** Cristina Aguado, Magdalena Gayà-Vidal, Sergi Villatoro, Meritxell Oliva, David Izquierdo, Carla Giner-Delgado, Víctor Montalvo, Judit García-González, Alexander Martínez-Fundichely, Laia Capilla, Aurora Ruiz-Herrera, Xavier Estivill, Marta Puig, Mario Cáceres

**Affiliations:** 1 Institut de Biotecnologia i de Biomedicina, Universitat Autònoma de Barcelona, Bellaterra (Barcelona), Spain; 2 Departament de Genètica i de Microbiologia, Universitat Autònoma de Barcelona, Bellaterra (Barcelona), Spain; 3 Departament de Biologia Celular, Fisiologia i Immunologia. Universitat Autònoma de Barcelona, Bellaterra (Barcelona), Spain; 4 Centre for Genomic Regulation (CRG), Barcelona, Spain; 5 Universitat Pompeu Fabra (UPF), Barcelona, Spain; 6 Institució Catalana de Recerca i Estudis Avançats (ICREA), Barcelona, Spain; Stanford University School of Medicine, United States of America

## Abstract

In recent years different types of structural variants (SVs) have been discovered in the human genome and their functional impact has become increasingly clear. Inversions, however, are poorly characterized and more difficult to study, especially those mediated by inverted repeats or segmental duplications. Here, we describe the results of a simple and fast inverse PCR (iPCR) protocol for high-throughput genotyping of a wide variety of inversions using a small amount of DNA. In particular, we analyzed 22 inversions predicted in humans ranging from 5.1 kb to 226 kb and mediated by inverted repeat sequences of 1.6–24 kb. First, we validated 17 of the 22 inversions in a panel of nine HapMap individuals from different populations, and we genotyped them in 68 additional individuals of European origin, with correct genetic transmission in ∼12 mother-father-child trios. Global inversion minor allele frequency varied between 1% and 49% and inversion genotypes were consistent with Hardy-Weinberg equilibrium. By analyzing the nucleotide variation and the haplotypes in these regions, we found that only four inversions have linked tag-SNPs and that in many cases there are multiple shared SNPs between standard and inverted chromosomes, suggesting an unexpected high degree of inversion recurrence during human evolution. iPCR was also used to check 16 of these inversions in four chimpanzees and two gorillas, and 10 showed both orientations either within or between species, providing additional support for their multiple origin. Finally, we have identified several inversions that include genes in the inverted or breakpoint regions, and at least one disrupts a potential coding gene. Thus, these results represent a significant advance in our understanding of inversion polymorphism in human populations and challenge the common view of a single origin of inversions, with important implications for inversion analysis in SNP-based studies.

## Author Summary

Inversions have been an evolutionary biology model for almost a century, and recently the discovery of a high amount of structural variation in multiple organisms, including humans, has renewed the interest in them. Since early on, it was shown that they were adaptive and that they were involved in human diseases. However, in humans, the study of inversions has lagged behind due to important limitations in the experimental methods to analyze them. Here, we have optimized a technique for high-throughput validation and genotyping of inversions mediated by inverted repeats. By genotyping 17 of these inversions in a diverse sample of human individuals, including many of European origin and several non-human primates, we have carried out the most complete genotyping effort of human inversions to date. The results of our study indicate that a high proportion of these inversions are recurrent and have occurred multiple times during evolution. This represents an example of the plasticity of the genome and opens a new paradigm in the study of inversions, challenging the common view that inversions have a unique origin.

## Introduction

Different types of structural variants (SVs), including deletions, duplications, insertions, inversions, and translocations, have been recently discovered in the human genome and there is growing evidence of their importance in human diseases, complex traits, and evolution [1]–[4]. Specifically, inversions, that involve a change of orientation of DNA, have been a model in evolutionary biology for almost 90 years and were shown to have adaptive effects in the genus *Drosophila* and other organisms [5], [6]. In humans, inversions have been associated to genetic diseases, such as Hemophilia A [7], and complex disorders, like asthma [8]. In addition, inversions could confer susceptibility to other rearrangements with negative phenotypic consequences, like the emerin gene deletion [9], [10], olfactory receptor-gene cluster translocation [11], and microdeletion syndromes such as those in 3q29 and 17q21.31 [12], [13]. Finally, human inversions could also have important evolutionary consequences, as in the case of the 17q21.31 region that has been related to increased female fertility and positive selection [14].

The majority of SV studies have been based on microarrays, which are very powerful to identify unbalanced changes like copy number variants (CNVs). Inversions, on the other hand, are very difficult to study because they do not usually result in gain or loss of DNA. Therefore, the knowledge of human inversions has lagged behind. This is reflected in the fact that although several hundred inversions have been reported in humans [4], few (<15) inversions have been characterized in greater detail [4], [11], [12], [14]–[22]. These and other studies [23]–[25] have shown that inversions are generated by two main processes: breaks in relatively simple regions that are joined in opposite orientation by non-homologous mechanisms or non-allelic homologous recombination (NAHR) between inverted repeats (IRs) (either repetitive elements or segmental duplications (SDs)). However, very little is known about the frequency and distribution of the inversions in human populations, with most studies limited to a handful of individuals. The main exceptions are the 17q21.31 inversion, in which the global distribution has been estimated by linkage with SNPs [14], six large inversions studied by FISH in 27 individuals of three populations [12], the worldwide genotyping of the 8p23 inversion distribution based on SNP data and genetic substructure [21], and the recent analysis of eight simple inversions in 42 human samples of diverse origins, including one inversion genotyped in 57 populations [20].

Traditional methods for inversion analysis include G-banding karyotyping [26], FISH [11], [12], Southern blot hybridization [9], or pulsed-field gel electrophoresis [19]. With the development of sequencing techniques, it has been possible to identify inversions at a genome-wide level by sequence comparison [18], [27], [28] or paired-end sequencing and mapping (PEM) [20], [23], [25], [29]–[32]. However, these techniques have important limitations. In most cases the presence of IRs at the inversion breakpoints hinders their detection using single reads or paired-end sequencing of short fragments, and PEM has a very high rate of false positives in inversion prediction [33], [34]. Moreover, all the above methods have a low throughput and can be applied only to a few individuals, providing just a partial picture of the distribution of the inversions. As an alternative, computational algorithms have been developed to take advantage of available nucleotide variation data in multiple individuals to predict inversions and estimate inversion genotypes [21], [35]–, although it is unclear how accurate these predictions are across populations. In addition, these algorithms work only for relatively large and frequent inversions. Thus, it is always necessary to validate inversion predictions using independent techniques and genotype them in a large number of individuals.

**Figure 1:**
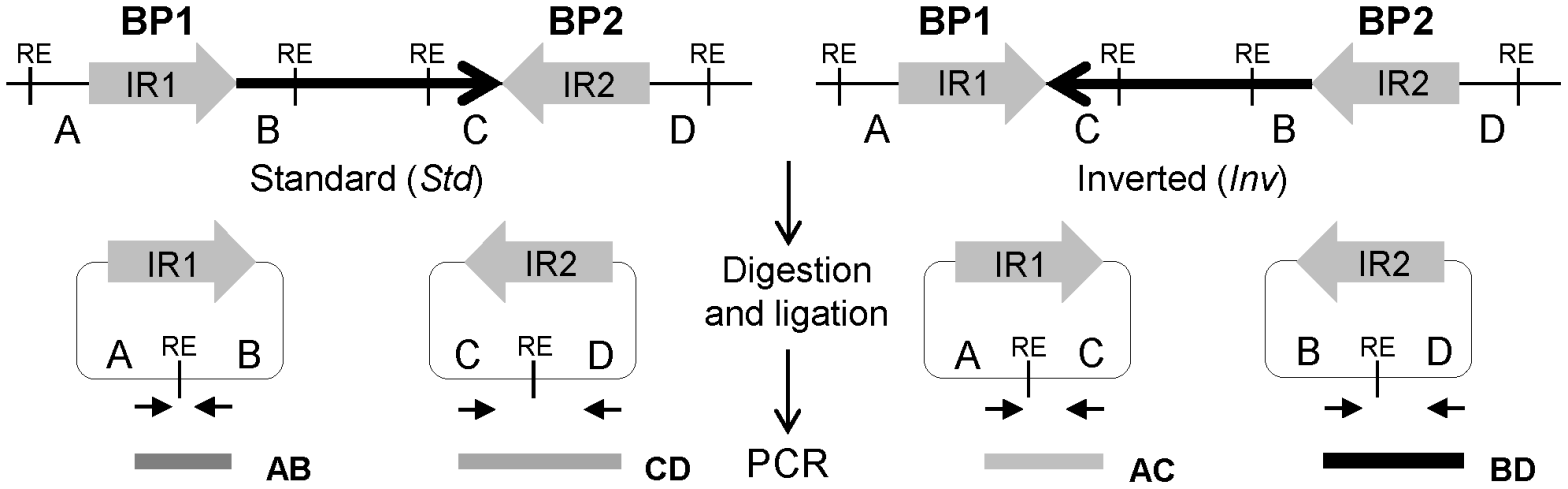
Diagram of iPCR validation of inversions mediated by inverted repeats (IRs). Standard and inverted arrangements are represented by unique regions A, B, C and D, which are separated by IR1 and IR2 at each inversion breakpoint (BP). The iPCR involves three steps: digestion at the restriction enzyme target sites (RE), circularization of the digested fragments by self-ligation, and PCR amplification with primers (small arrows) flanking the restriction site to generate products of different size depending on the orientation of the segment between both repeats.

PCR amplification offers more possibilities for high-throughput analysis and different PCR-based techniques have been used to validate inversions. Regular or long-range PCR [20], [25], [27], [38], [39] are limited by the size of the fragments to amplify and work poorly for fragments above 10 kb. Therefore, their applicability is reduced to inversions generated by simple breaks or small IRs at their breakpoints. Haplotype-fusion PCR is a very promising technique to study inversions caused by duplicated sequences of almost any kind [40], [41], although it has not yet been used extensively and reproducibly to genotype inversions. Inverse PCR (iPCR) [42] is based on creating circular molecules of DNA by restriction enzyme digestion and self-ligation, followed by amplification across a self-ligated site (Figure 1). Thus, with iPCR there is no need to amplify across the breakpoints and it is possible to analyze inversions mediated by medium-long IRs. iPCR has been used to sequence the flanking regions of known sequences [43], sequence breakpoints of translocations [44], [45], or generate long insert pairs [46]. In addition, an iPCR assay has been applied to genotype inversions mediated by 9.5 kb SDs causing hemophilia A in multiple patients [47]–[50] and in prenatal diagnosis [51]. However, iPCR limits for different types of inversions have not yet been tested. Therefore, optimization of the iPCR technique could open the possibility of high-throughput validation and genotyping of inversions in a simple manner.

Here, we have tested different reagents and conditions to optimize the iPCR method and developed a high-throughput iPCR protocol that allows us to genotype a wide-variety of inversions mediated by IRs in a large number of individuals in just one day. As an example of the potential use of this method, we have analyzed 22 inversions predicted in the human genome by PEM and determined the frequency and genetic transmission of 17 of them in 70 CEU individuals. In addition, we have checked the association between inversions and nucleotide variation using the HapMap [52] and 1000 Genomes Project (1000GP) [53] SNP data, the putative ancestral orientation, and the possible gene effects of the validated inversions. All this information has been deposited in the new InvFEST human polymorphic inversion database [54].

## Results

### Selection of candidate polymorphic inversions

Inversion validation was based on the analysis of PEM data from fosmids of nine different individuals [23] with our inversion prediction algorithm GRIAL to confirm the presence of an inversion signature and refine the breakpoint locations [54]. For simplicity, we always refer to the orientation matching the NCBI36 (HG18) human reference genome as standard (*Std*) and the opposite orientation as inverted (*Inv*) arrangement. We selected 31 inversion candidates present in at least one of the nine individuals with ≥2 fosmids supporting both the *Std* and *Inv* orientations, and IRs of <40 kb in the predicted ranges of the two breakpoints. The next step was finding restriction enzymes that cut at both sides of the inversion breakpoints, but not within the IRs, and that generate fragments of less than ∼70 kb. This way we identified 22 potential inversions for iPCR analysis distributed in 11 chromosomes and with a size of 5.1–226 kb (Table 1 and Table S1). For 13 inversions, the region of sequence exchange could be defined more precisely from at least one completely sequenced BAC or fosmid showing the inverted conformation [23], [24]. In all cases the refined breakpoints were located either within or at the ends of the IRs in a region of high sequence identity between them [54]. Finally, we compared the selected regions in HG18 with those in the GRCh37 (HG19) assembly or additional genome patches [55] and in three cases we found important changes between them, which were analyzed in more detail (see below).

**Table 1:** Summary of the characteristics of the analyzed inversion predictions and iPCR validation results.

Inversion	Chr.	Breakpoint 1 (NCBI36/HG18)	Breakpoint 2 (NCBI36/HG18)	Inverted repeat (IR)	IR1/IR2 size (kb)	Inversion size^1^ (kb)	Fragments iPCR (kb)	Validation status	iPCR/PEM consistency^2^
HsInv0114	chr9	125778473–125781206	125793139–125795872	TEs/SDs^6^	2.7/2.7	14.7	13.8–17.8	Inversion	Yes (9/9)
HsInv0124	chr11	300225–301836	307945–309555	SDs	4.5/8.9	7.7	2.3–14.8	Inversion	Yes (9/9)
HsInv0209	chr11	70953485–70960513^5^	70965419–70972446^5^	SDs	7.0/7.0	11.9	9.2–11.7	Inversion	Yes (9/9)
HsInv0241	chr2	241264234–241270541	241280391–241286847	SDs	13.3/12.8	16.2	17–20.1	Inversion	Yes (7/7)
HsInv0272	chr5	178802777–178811616^5^	178857371–178867555^5^	SDs	8.8/10.2	55.3	8.8–17.6	Not validated	Yes (9/9)
HsInv0278	chr5	180455356–180458186^5^	180460457–180463248^5^	SDs	2.8/2.8	5.1	3.3–13.8	Inversion	Yes (8/8)
HsInv0286	chr7	54258468–54259261	54353522–54354315	TEs/SDs^7^	15.9/15.9	95.1	23.3–41.5	Inversion	Yes (9/9)
HsInv0306^3^	chr8	2272127–2276815^5^	2311945–2316621^5^	L1PA7, AluSc	4.7/4.7	39.8	9.7–25.6	Complex region	-
HsInv0340	chr13	63188985–63193242	63237334–63241591	SDs	10.6/10.6	48.4	31.6–39.9	Inversion	Yes (9/9)
HsInv0341	chr13	79291903–79294413	79312733–79315243	L1PA3	6.2/6.2	20.8	7.2–17.1	Inversion	Yes (9/9)
HsInv0344	chr14	34079802–34086813	34094204–34101227	SDs	7.2/7.2	14.4	9.8–28.7	Inversion	Yes (9/9)
HsInv0347	chr14	60141001–60142582	60148138–60149719	THE1C	1.6/1.6	7.1	4.3–9.2	Inversion	Yes (8/8)
HsInv0389	chrX	153219602–153228808	153266422–153275629	SDs	11.3/11.3	46.8	12–30.8	Inversion	Yes (9/9)
HsInv0393	chrX	100739178–100743833	100753236–100757891	SDs	4.7/4.7	14.1	7.3–8.5	Inversion	Yes (9/9)
HsInv0396	chrX	72132652–72141803	72214354–72223499	SDs	9.5/9.5	81.7	12.3–17.9	Inversion	Yes (9/9)
HsInv0397	chrX	105396369–105408238	105417867–105429736	SDs	32.2/15.1	21.5	18.4–20.7	Inversion	Yes (8/8)
HsInv0403^4^	chrX	75278106–75282269	75285318–75289479	TEs/SDs^8^	4.3/4.3	7.2	8.2–10.1	Inversion	Yes (5/5)
HsInv0414	chrX	151985038–151993457^5^	151994653–152003088^5^	SDs	8.4/8.4	9.6	-	Complex region	-
HsInv0526	chr11	116509421–116514763^5^	116581672–116587255^5^	SDs	5.3/5.6	72.4	9.1–27.6	Not validated	Yes (9/9)
HsInv0710^3^	chr8	2167585–2182892^5^	2316618–2331389^5^	SDs	15.3/14.8	148.8	25.6–33.2	Complex region	-
HsInv0832	chrY	16496132–16504854^5^	16517493–16526218^5^	SDs	8.7/8.7	21.4	15.5–37.4	Inversion	Yes (1/1)
HsInv1051	chr17	18442024–18466271^5^	18667812–18692134^5^	SDs	24.2/24.3	225.8	46.4–72.7	Inversion	Yes (9/9)

1 Inversion size is calculated as the distance between the middle positions of the two breakpoint intervals.

2 Consistency between the iPCR and paired-end mapping (PEM) results is indicated by the number of individuals in which the iPCR genotype is consistent with the presence of fosmids supporting the *Std* or *Inv* orientation after excluding those mapping within the IRs (see Table S2).

3 HsInv0306 includes this inversion prediction and HsInv0312, whereas HsInv0710 includes this inversion prediction and HsInv0311 [54].

4 The inverted region of inversion HsInv0403 shows the opposite orientation in the GRCh37 (HG19) assembly.

5 For inversion predictions in which no inverted sequence was available, breakpoint coordinates correspond to the whole length of the segmental duplications (SDs) annotated in HG18 or the IRs defined by BLAST alignment of the two breakpoints.

6 IRs include sequences of transposable elements (TEs) L2, MLT1K, MLT1C, AluSq, L3, and MIRb, although the whole region has been anotated as SD in HG19.

7 IRs are annotated as SDs in HG18, but not in HG19 since they are completely formed by multiple partial copies of different TEs.

8 IRs are not annotated as SDs in HG18 or HG19, but contain unique sequences and parts of different TEs (L2, L4, MER41C, MER4A, and MamGyp).

### iPCR optimization

Figure 1 shows the general iPCR design for inversions mediated by IRs between regions A and B (breakpoint 1, BP1) and C and D (breakpoint 2, BP2). Previous studies had shown that iPCR could be used to validate inversions mediated by IRs with self-ligating circular fragments of up to 21.5 kb [47]. Therefore, to apply this technique to the largest range of inversions possible, we first tested the optimal DNA concentration and the effects of different reagents in the circularization efficiency of long DNA fragments. The analysis was based on the previous work of Wo et al. [56], and we selected two inversions, HsInv0340 and HsInv0286, which generate circular molecules of approximately 30–40 kb in the two orientations when cut with the staggered-end enzyme *BamHI* or the blunt-end enzyme *SwaI*.

**Figure 2:**
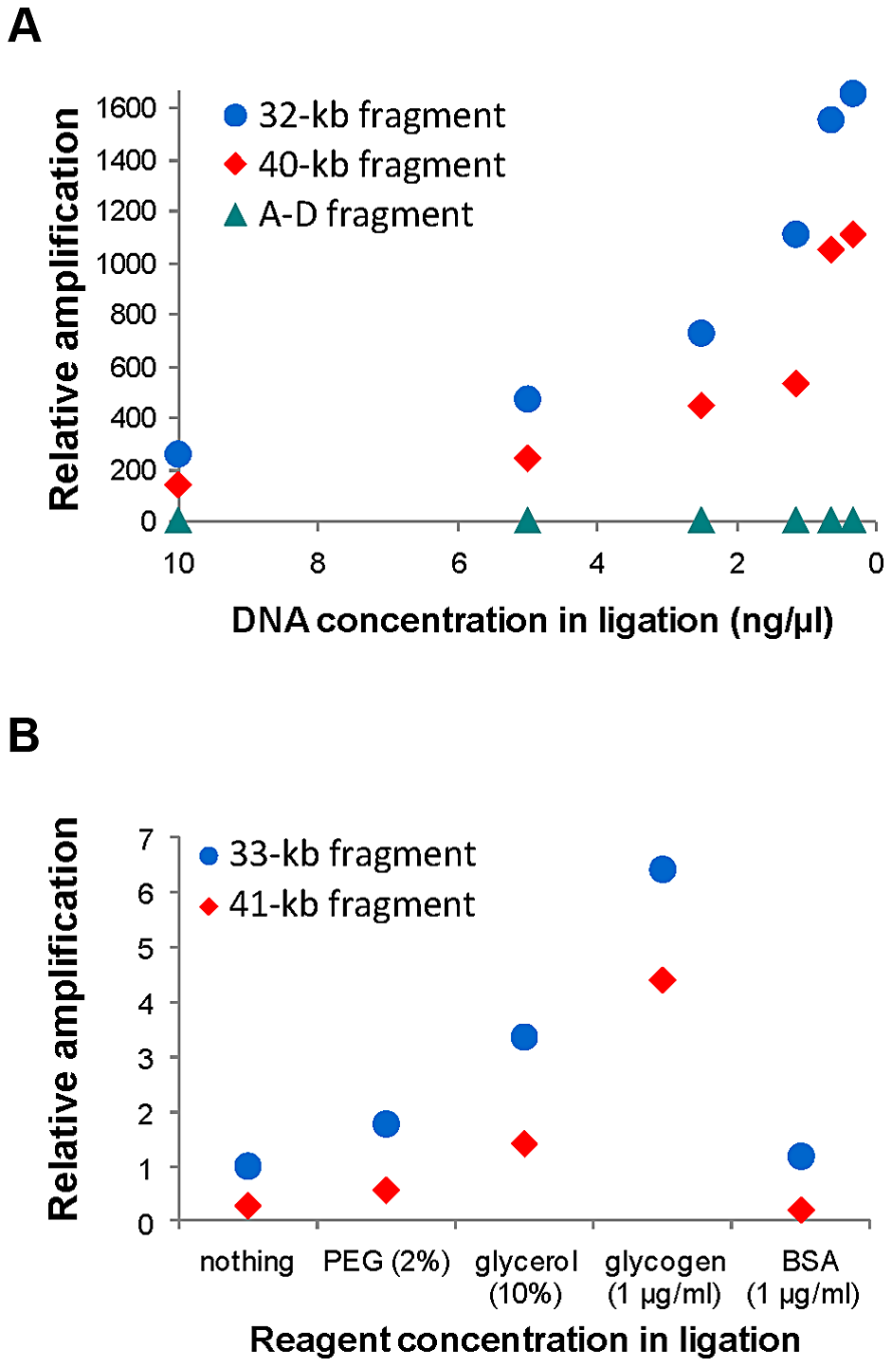
Quantification of self-ligation efficiency in iPCR with different DNA dilutions and different reagents. **A**. Effect of DNA dilution during ligation on iPCR amplification of staggered-end fragments from HsInv0340 of 32 kb (AB) and 40 kb (BD) and the A–D concatamer. **B**. Effect of different reagent concentrations during ligation on iPCR amplification of blunt-end fragments from HsInv0286 of 33 kb (BD) and 41 kb (AB). The A–D fragment with 10 ng/µl of DNA and the 41 kb fragment with no additional reagent were selected as reference to calculate the relative amplification in **A** and **B**, respectively. NA18517 DNA that is heterozygous for both inversions was used in the analysis.

Consistent with previous observations [56], for the inversion analyzed with a staggered-end enzyme (HsInv0340), we found a continuous increase in iPCR amplification with DNA dilution during ligation, with the most diluted DNA (0.31 ng/µl) showing a 6.5–8.2-fold improvement in circularization efficiency for both sizes of DNA circles (Figure 2A). For the inversion analyzed by iPCR with blunt ends (HsInv0286), we observed a similar trend for the two fragment sizes, although amplification was too low for accurate quantification. Next, using one of the optimal DNA dilutions (0.62 ng/µl), we found that the addition of different reagents did not produce an improvement in the self-ligation step of staggered-end fragments. In blunt-end fragments there was an increase in amplification when PEG, glycerol and glycogen were used (Figure 2B). In particular, glycogen showed a 6.4–15.7-fold increase in the self-ligation of 33 kb and 41 kb fragments. Also, we quantified the possibility that the iPCR target region is produced by ligation between two different fragments using as a control the AD product, which could be generated only by the formation of concatamers. However, AD amplification was nonexistent from blunt-end fragments and very low and constant throughout the dilutions and the reagents tested for staggered-end fragments (Figure 2).

### Validation of predicted inversions by iPCR

**Figure 3:**
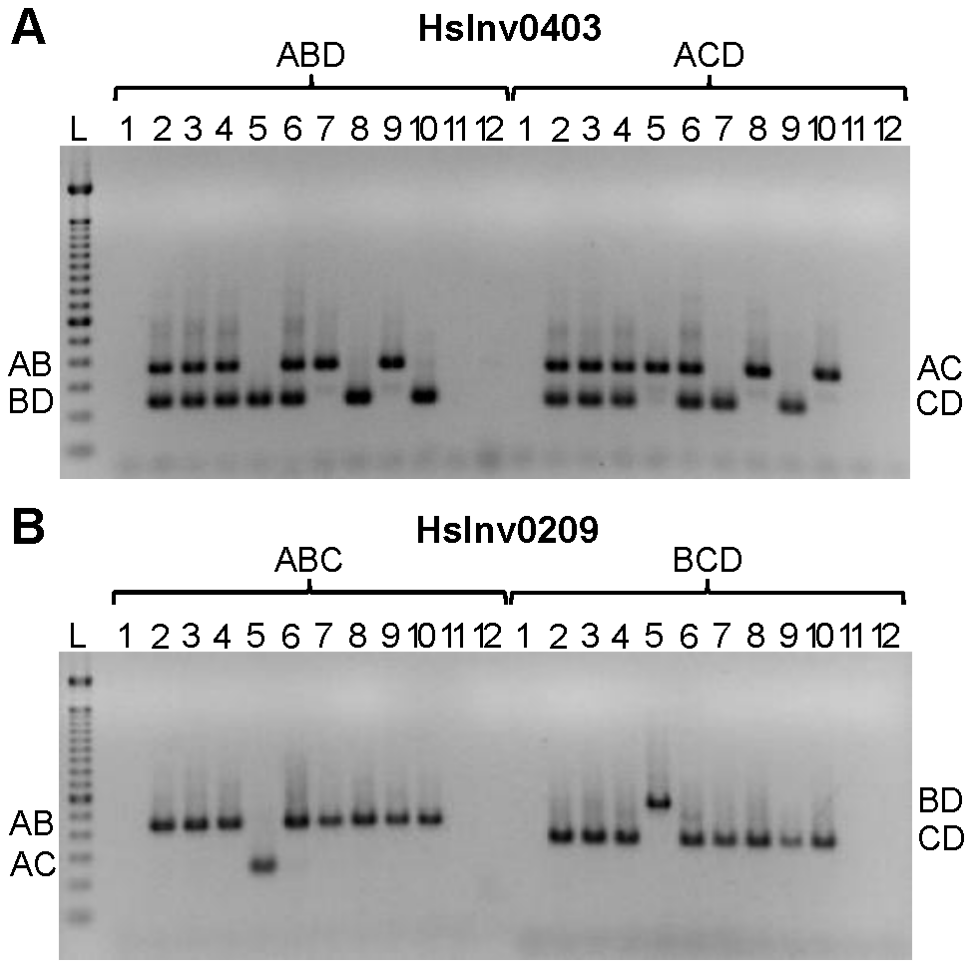
Multiplex iPCR results of two validated inversions in nine human samples. **A**. HsInv0403 ABD and ACD iPCRs. Band sizes are: AB, 364 bp; BD, 239 bp; AC, 350 bp; CD, 225 bp; and AD, 265 bp. **B**. HsInv0209 ABC and BCD iPCRs. Band sizes are AB, 435 bp; AC, 243 bp; BD, 543 bp; CD, 351 bp; and BC, 470 bp. For both panels the genomic DNA samples are: 1, negative control; 2, NA12156; 3, NA12878; 4, NA15510; 5, NA18507; 6, NA18517; 7, NA18555; 8, NA18956; 9, NA19129; 10, NA19240; 11, DNA without restriction enzyme; 12, DNA without T4 DNA ligase; and L, 100 bp DNA Ladder (Invitrogen).

Based on the results obtained in the optimization experiments, we developed iPCR assays to validate the two breakpoints of the previously selected inversions, except for the three regions not correctly assembled in the HG18 sequence. For the remaining 19 inversions, the restriction enzyme target site and the primers were selected outside of the refined breakpoint regions involved in the inversion (Table S1), although in three cases (HsInv0124, HsInv0397, and HsInv0272) one of the primers and restriction sites were located in a divergent region between the two IRs. Overall, we used seven different enzymes and the size of the self-ligation fragments varied between 2.3 and 73 kb (Table 1 and Table S1). As a first validation step, for all inversions we did multiplex iPCRs of both potential orientations of the two breakpoints (AB or CD fragments and AC or BD fragments) in the nine individuals of diverse origins used to predict the inversions by PEM [23]. Figure 3 shows the amplification results of two inversions as an example. We did not observe the expected bands in undigested or unligated genomic DNA controls in any inversion. For many of the iPCR assays we also tested the amplification of the AD fragment concatamer, and no or very little amplification was found.

The iPCR genotypes from both breakpoints were the same in 16 of the 19 inversions (Table S2), but in the other three there were some discrepancies. In HsInv1051 there is no BD amplification in the only individual heterozygous for the inversion according to the fosmids and the AC iPCR (NA19240; Table S2), although this case is particularly challenging because it corresponds to the largest self-annealing circle we have tried to amplify (73 kb). Similarly, in HsInv0397 there is one individual (NA12156) with different genotype for BP1 (*Std/Inv*) and BP2 (*Std/Std*). However, according to the fosmid information the individual should be heterozygous for the inversion (Table S2). In HsInv0241 three samples appear as *Std/Inv* in BP1 and *Inv/Inv* in BP2, and the same results were found with a new set of primers for the CD region. By genotyping known SNPs within the HsInv0241 inverted region through sequencing and restriction enzyme digestion, we showed that an allele is missing in BP2 in some of the supposedly *Inv* homozygous individuals (data not shown). Therefore, for the last two inversions it is likely that there is an unknown structural variant or restriction site polymorphism (either a new restriction site or the elimination of the restriction site used in the iPCR) that prevents BP2 amplification in one orientation, and that the assay showing the two chromosome arrangements represents the correct genotype. Furthermore, to assess the reproducibility of this technique, for inversions HsInv0344 and HsInv0389 we showed that the same iPCR results were obtained with two different restriction enzymes (*BamHI* or *EcoRI* for HsInv0344 and *NsiI* or *BglII* for HsInv0389). Thus, with a good design and the appropriate controls, inversion genotyping by iPCR is quite robust.

With regard to the inversion validation, in 17 of the 19 inversions the two genomic orientations were found in the panel of nine humans, but in four of them the *Inv* arrangement was present only in one of the original Yoruban (YRI) individuals used to predict the inversion (Table S2). In the remaining two inversions (HsInv0272 and HsInv0526), iPCR of both breakpoints only identified the *Std* arrangement in all individuals and are likely false positives in the PEM analysis. To check the validity of iPCR results, we compared them with those of the fosmid PEM [23]. To do this, for each individual we considered only the fosmids whose ends map uniquely and outside the IRs at the breakpoints, and those mapping within the IRs were discarded since they are often not informative. For the 17 validated inversions, the genotypes obtained with iPCR and the fosmid PEM data are consistent for all individuals (Table S2). The false positive inversion predictions HsInv0272 and HsInv0526 were supported just by a few fosmids and all of them have one read mapping in a particular region within the inverted SDs. Sequence analysis has shown that in both cases the incorrect fosmid mappings are caused by variation across individuals in the nucleotide divergence between the two SD copies.

### Analysis of inversion predictions in complex genomic regions

For three of the selected candidates in HG18, HsInv0306, HsInv0414, and HsInv0710, the genomic regions show a different and more complex organization in subsequent releases of the human genome sequence. Therefore, we re-evaluated the inversion predictions taking into account the new data and the preliminary iPCR results. In HsInv0414, the whole region was duplicated in the HG19 assembly and a 50-kb gap was created, which makes the design and the interpretation of iPCRs very difficult and the region was not analyzed any further.

HsInv0710 and HsInv0306 are two overlapping inversion predictions of different length supported by many discordant fosmids. This region has been updated with a new sequence patch (GL949743.1), which has 75 kb of extra sequence that transforms the 15-kb inverted SDs found in HG18 and HG19 into two SD blocks of 109 kb and 95 kb (Figure S1). To determine if the two inversion candidates are still valid in this context, we re-mapped a set of 1725 concordant and discordant fosmid paired-end reads with mappings spanning the region of interest +/−50 kb (see Materials and Methods). We obtained a total of 20 discordant paired-end reads, and most of the fosmids originally supporting the inversion predictions mapped in highly identical regions within the new SD blocks and were not informative. For HsInv0710, only one of the 53 fosmids still supports the inversion, although it maps within the inverted SDs with just slightly higher score in the discordant than the concordant orientation. For HsInv0306, 19 of the 65 fosmids continue to map as discordant in orientation in the HG19 patch (Figure S1). However, a similar amount of fosmids from all the individuals also support the reference orientation and the 19 apparently discordant fosmids are explained by a ∼16 kb polymorphic deletion of part of SD2 (Figure S1). Thus, there is not reliable PEM evidence that these inversions exist. Due to the size of the new SDs it was not possible to interrogate the presence of the inversion by iPCR. Nevertheless, we designed several iPCR primers to confirm the organization of the genomic region (Figure S1). For HsInv0710, six of the nine individuals are heterozygous for AB and BD (the other three being homozygous for AB) and the fragments AC and CD were never amplified. Similarly, for HsInv0306, the nine individuals showed AB and AC amplification. These results support the existence of big SDs and indicate that the sequence of the new patch is probably correct.

### Genotyping of validated inversions in multiple individuals

In order to genotype inversions in multiple individuals, we set up the iPCR protocol in a plate format. For each of the 17 true polymorphic inversions, we analyzed one breakpoint in 77 individuals, including 68 additional CEPH samples with ancestry from northern and western Europe (CEU) and 12 father-mother-child trios. The genotyping worked very well, with on average more than 98% valid genotypes for iPCRs with staggered-end enzymes (Table S3). For six inversions, we repeated 40–100% of the individuals in independent iPCRs testing the other breakpoint and in all cases the results were the same. The only exception was inversion HsInv0241, in which both breakpoints were tested for all individuals, and as in the previous experiments, 23 individuals (30%) showed different results in BP1 (*Std/Std* or *Std/Inv*) and BP2 (nothing or *Inv/Inv*). As mentioned before, these results are consistent with a missing *Std* allele in the iPCR of BP2 in these individuals and we considered only the genotypes obtained from AB and BD amplifications. In addition, for inversion HsInv0286, which involved blunt ends, there was no clear amplification in some DNAs and we could obtain reliable genotypes for 48 CEU individuals (Table S3).

Inversion frequency and heterozygosity on the CEU population varied considerably (Table 2). Of the 17 inversions, two (HsInv0832 and HsInv1051) were not present in any of the CEU individuals, three (HsInv0209, HsInv0340, and HsInv0341) showed very low frequency (<5%), and the rest had a minor allele frequency (MAF) between 10–47% (Table 2). Likewise, the observed heterozygosities (H) ranged from 0 to 0.55, and all polymorphic inversions were in Hardy-Weinberg equilibrium (Table 2). As a control, we also analyzed the genetic transmission of the *Std* and *Inv* allele in the available family trios, and in all cases it fits perfectly the expected inheritance pattern (Table 2). For inversions HsInv0209, HsInv0340, HsInv0341, HsInv0832 and HsInv1051 we could not analyze the inheritance for the *Inv* allele in the CEU population. Thus, we checked the families of the YRI individuals with the inversion and in all of them the *Inv* allele was correctly transmitted (Table S3), showing that the inversion genotypes obtained by iPCR behave as a normal genetic variant.

**Table 2:** Summary information of the genotyping in CEU individuals, gene effect, and evolutionary history for the 17 validated polymorphic inversions in the human genome.

Inversion	Chr.	N^1^	*Inv* freq.	Observed heterozygosity	Valid families	Genes affected		iPCR results^2^		Ancestral orientation^3^
						Breakpoints	Inverted region	Chimp	Gorilla	
HsInv0114	chr9	92	0.64	0.50	12/12	no	no	*Inv*	Inv*	*Inv*
HsInv0124	chr11	92	0.39	0.43	12/12	*BC040735*	*IFITM1*	*Std*	*Std*	*Std*
HsInv0209	chr11	90	0.02	0.04	12/12	*KRTAP5-10, KRTAP5-11*	no	*Std*	*Std*	***Std***
HsInv0241	chr2	92	0.16	0.28	12/12	*AQP12A, AQP12B*	no	*Std*	*Std/Inv*	***Std***
HsInv0278	chr5	92	0.10	0.20	12/12	*TRNA_Val, TRNA_Leu*	no	*Inv*	Inv*	*Inv*
HsInv0286	chr7	72	0.47	0.50	3/3	no	no	*Std*	*Std*	***Std***
HsInv0340	chr13	90	0.01	0.02	11/11	no	*OR7E156P, LOC647264*	*Std/Inv*	Inv*	*Inv*
HsInv0341	chr13	92	0.03	0.07	12/12	no	no	*Std/Inv*	*Std*	***Std***
HsInv0344	chr14	92	0.59	0.48	12/12	*SNX6*	no	*Std/Inv*	ND	Unknown
HsInv0347	chr14	92	0.10	0.15	12/12	no	no	*Std*	*Std/Inv*	*Std*
HsInv0389	chrX	69/46	0.17	0.17	14/14	no	*FLNA, EMD*	*Inv*	Inv*	*Inv*
HsInv0393	chrX	66/44	0.36	0.55	13/13	*ARMCX6*	no	*Std/Inv*	*Inv*	***Inv***
HsInv0396	chrX	69/46	0.16	0.22	14/14	*PABPC1L2A, PABPC1L2B*	no	*Std/Inv*	*Std*	***Std***
HsInv0397	chrX	68/46	0.18	0.35	13/13	no	no	*Std/Inv*	Inv*	*Inv*
HsInv0403	chrX	69/46	0.26	0.30	14/14	no	no	*Std/Inv*	*Std/Inv*	Unknown
HsInv0832	chrY	25	0	0	7/7	no	no	*Inv*	*Std*	Unknown
HsInv1051	chr17	86	0	0	9/9	*CCDC144B*	7 genes^4^	ND	ND	*Std*

1 N, number of chromosomes from unrelated individuals.

For inversions located on chr. X, the first number refers to the chromosomes used to calculate the frequency of the inversion and the second number to the chromosomes in females used to calculate the heterozygosity.

2 iPCR results derive from the analysis of four chimpanzees and two gorillas and those not coinciding with the current orientation of the species genome (panTro4 or gorGor3) are marked with an asterisk.

For HsInv0389, iPCR data from chimpanzees is consistent with the experimental analysis of Cáceres et al. [59]. ND, not determined.

3 Estimation of the ancestral orientation is based mainly on the iPCR results for chimpanzee and gorilla.

Those cases in which the phylogenetic trees are informative and support the results from the iPCR are shown in boldface. For HsInv1051, the ancestral state is based on the chimpanzee genome and the disruption of the *CCDC144B* gene in the inverted orientation.

4 Genes completely included within the inversion are *TBC1D28*, *ZNF286B*, *FOXO3B*, *TRIM16L*, *FBXW10*, *FAM18B*1, and *DKFZp434O1826*.

### Association of inversions and nucleotide variation

Inversions are known to inhibit recombination and generate genetic substructure with high linkage disequilibrium (LD) of the SNPs within the inverted region [5], [6]. Therefore, we examined the patterns of nucleotide variation within the inverted region and in the 10 kb flanking the 14 inversions with at least two inverted alleles in the genotyped CEU individuals using the SNP data from HapMap (46 unrelated individuals) [52] and 1000GP (28 unrelated individuals) [53]. The HapMap data revealed perfect LD (*r^2^* = 1) between the inversion HsInv0286 and HsInv0347 and several SNPs (Table 3 and Table S4). In addition, inversion HsInv0396 presented very high LD values (*r^2^* = 0.9), with three SNPs. The 1000GP data allowed us to detect more SNPs with perfect LD for the three inversions mentioned above and HsInv0114 (Table 3 and Table S4). As expected, most of these tag SNPs were located inside the inverted region, although some of them were located outside as well (Table 3).

**Table 3:** Nucleotide variation data from HapMap and 1000 Genomes Project (1000GP) for 14 polymorphic inversions with >2 inverted chromosomes in the CEU population.

	HapMap genotypes				1000GP genotypes				1000GP haplotypes				
Inversion	N^1^	SNPs	Fixed	Shared	N^1^	SNPs	Fixed	Shared	Sites	Fst^2^	π_all_	π*_Std_*	π*_Inv_*
*Inverted region* 3													
HsInv0114	46	16	0	1	28	43	2	2	11932	**0.63** *	0.00064	0.00061	0.00020
HsInv0124	46	2	0	1	28	18	0	6	6109	**0.31** *	0.00066	0.00065	0.00039
HsInv0209	45	6	0	1	28	17	0	0	4906	0.41	0.00095	0.00093	-
HsInv0241	46	3	0	0	28	10	0	0	3178	**0.28** *	0.00098	0.00093	0.00063
HsInv0278	46	2	0	2	28	17	0	0	2271	**0.32** *	0.00258	0.00255	0.00053
HsInv0286	33	51	1	3	21	393	5	9	74342	**0.45** *	0.00077	0.00087	0.00035
HsInv0341	45	19	0	12	28	55	0	28	16881	0.00	0.00101	0.00101	0.00122
HsInv0344	46	3	0	1	28	36	0	20	7368	**0.31** *	0.00095	0.00069	0.00086
HsInv0347	46	3	2	0	28	11	2	0	5543	**0.82** *	0.00025	0.00011	0.00036
HsInv0389	46	16	0	14	28	60	0	35	37610	**0.70** *	0.00030	0.00015	0.00034
HsInv0393	44	3	0	3	27	16	0	4	9399	**0.59** *	0.00030	0.00014	0.00025
HsInv0396	46	29	0	0	28	192	12	5	71906	**0.65** *	0.00081	0.00060	0.00004
HsInv0397	45	0	0	0	27	3	0	0	8906	0.00	0.00001	0.00001	0.00000
HsInv0403	46	1	0	1	28	5	0	5	2796	**0.24** *	0.00050	0.00056	0.00039
*Flanking region* 4													
HsInv0114	46	14	0	4	28	80	2	12	19949	**0.62** *	0.00071	0.00053	0.00030
HsInv0124	46	16	0	9	28	110	0	43	19999	**0.32** *	0.00141	0.00108	0.00121
HsInv0209	45	24	0	0	28	106	0	0	19998	0.16	0.00164	0.00163	-
HsInv0241	46	14	0	3	28	60	0	7	19999	**0.25** *	0.00098	0.00099	0.00036
HsInv0278	46	17	0	9	28	95	0	7	19993	**0.24** *	0.00121	0.00119	0.00074
HsInv0286	33	11	1	0	21	121	1	2	19987	**0.39** *	0.00082	0.00095	0.00038
HsInv0341	45	23	0	10	28	61	0	16	19992	0.00	0.00099	0.00100	0.00107
HsInv0344	46	11	0	10	28	73	0	35	19991	**0.30** *	0.00081	0.00064	0.00069
HsInv0347	46	14	5	0	28	32	10	2	19999	**0.95** *	0.00020	0.00002	0.00033
HsInv0389	45	19	0	17	28	34	0	22	20000	**0.50** *	0.00031	0.00019	0.00048
HsInv0393	43	13	0	7	27	32	0	10	19996	**0.46** *	0.00039	0.00022	0.00042
HsInv0396	45	10	0	0	28	42	1	1	19998	**0.60** *	0.00049	0.00037	0.00010
HsInv0397	45	1	0	1	27	15	0	1	20000	**0.22** *	0.00008	0.00008	0.00003
HsInv0403	46	9	0	0	28	58	0	18	20000	**0.62** *	0.00088	0.00045	0.00064

1 N, number of unrelated individuals analyzed.

2 Fst values were calculated comparing the *Inv* and *Std* inferred haplotypes.

* *P*<0.05.

3 The inverted region includes the region within the IRs that mediated the inversion.

4 The flanking region corresponds to 10 kb outside each IR.

Genotype data were also used to calculate the minimum number of shared SNPs between the two arrangements without phase estimation. To minimize the effect of SNP genotyping errors, for the 1000GP data we performed these calculations using all genotypes and only the most reliable ones based on the genotype likelihoods. Surprisingly, for six inversions (HsInv0124, HsInv0341, HsInv0344, HsInv0389, HsInv0393, HsInv0403) a high proportion of the HapMap and 1000GP SNPs located within the inversion were shared between the two arrangements (Table 3). That is, according to the genotype information, these nucleotide variants were polymorphic both in *Std* and *Inv* chromosomes, and the shared SNPs were distributed over the whole inverted region. In contrast, a much lower proportion of shared SNPs between arrangements was observed for the four inversions (HsInv0114, HsInv0286, HsInv0347, HsInv0396) for which SNPs in high LD were identified (Table 3). The remaining inversions did not present such a clear pattern, mainly due to either a very low frequency of the inversion (HsInv0278, HsInv0209) or a low number of SNPs within the region (HsInv0241, HsInv0397). Very similar results were observed when using only the filtered most-reliable 1000GP SNP genotypes (Table S5).

**Figure 4:**
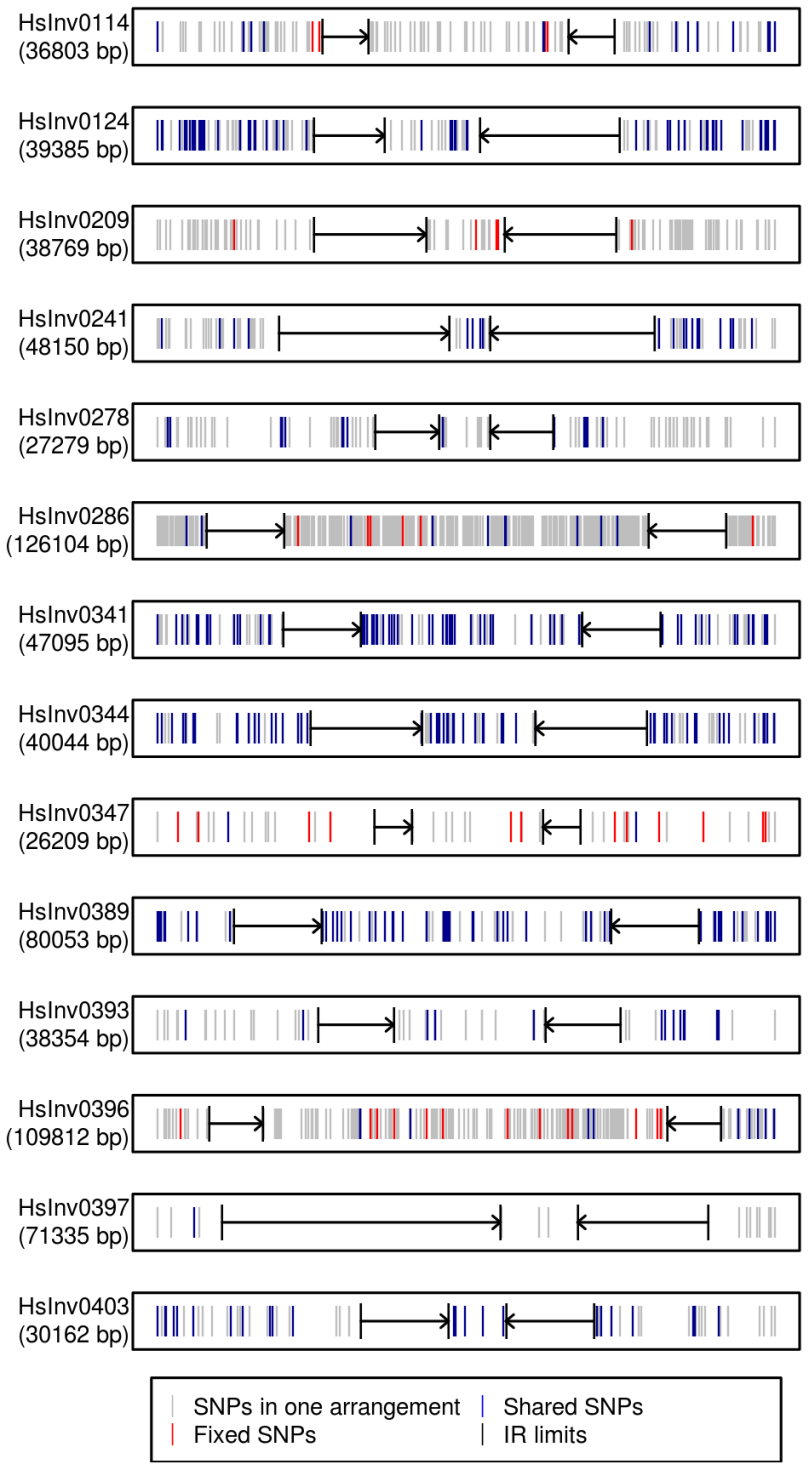
Distribution of SNPs along 14 polymorphic inversions in CEU individuals. SNP distribution was calculated according to the haplotypes inferred from the 1000 Genomes Project data by PHASE. Both the inverted region and 10(indicated by black arrows) are represented. Polymorphic SNPs in the *Std* or *Inv* arrangement are shown in grey, fixed SNPs between arrangements are shown in red, and shared SNPs between arrangements are shown in blue.

Next, by inferring haplotypes using the PHASE program [57], [58], we were able to calculate the nucleotide diversity (π) in *Std* and *Inv* chromosomes using the 1000GP data. For most inversions, π values were similar in both arrangements, although for HsInv0278 and HsInv0396, π of *Inv* chromosomes was more than five times lower (Table 3). In addition, we used Fst to measure genetic differentiation between arrangements (Table 3). In general, the most significant Fst values were observed for the inversions with tag SNPs. An exception was HsInv0389, in which the differentiation is due to the fact that there were two sets of very different haplotypes, that were mainly *Std* or *Inv*, although some haplotypes were shared between arrangements. On the contrary, Fst values of the inversions with shared SNPs tended to be low or even zero, consistent with the absence or very small differentiation between orientations. Figure 4 shows the distribution of fixed and shared SNPs along the inverted region.

### Evolutionary history of the inversions

**Figure 5:**
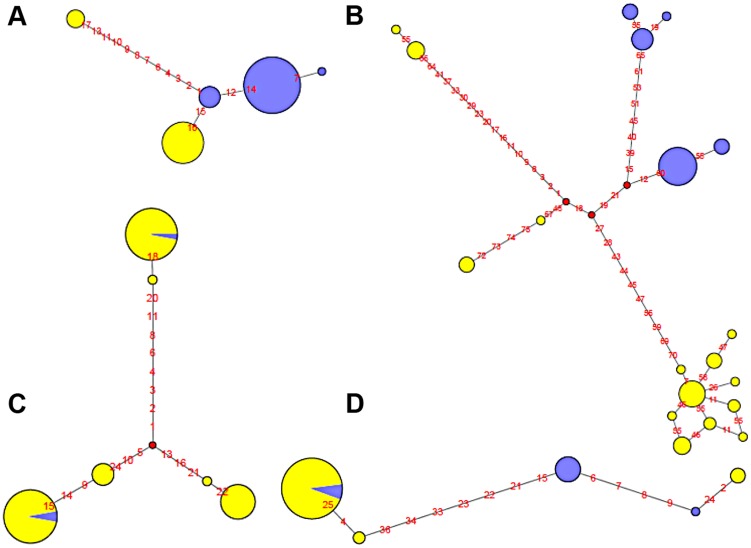
Haplotype network of four human polymorphic inversions from phased HapMap SNP data showing unique or recurrent origins. **A**. HsInv0114. **B**. HsInv0286. **C**. HsInv0341. **D**. HsInv0389. Circles correspond to the different haplotypes found for the region of the inversion and circle sizes are proportional to the frequency of each haplotype. *Std* and *Inv* haplotypes are represented in yellow and blue, respectively. Small red circles represent hypothetical haplotypes. Nucleotide changes between haplotypes are indicated as red numbers.

To illustrate the relationship between phased haplotypes within the inverted region, Median-Joining networks and Neighbor-Joining trees based on HapMap and 1000GP SNPs were constructed for all inversions (Figure 5 and Figure S2). The 14 inversions could be classified into three main groups. For the four inversions with fixed SNPs between arrangements (HsInv0114, HsInv0286, HsInv0347, HsInv0396), the networks and trees showed that the *Std* and *Inv* haplotypes formed separate clusters (Figure 5A and 5B), suggesting that the inversion arose from a unique event. In the case of HsInv0124, the haplotype network derived from the 1000GP data shows many recombination events, including possible gene conversion between arrangements. However, the network of the inverted and flanking regions (+/−10 kb) from the HapMap data (not shown) has two clearly separated clusters of *Std* and *Inv* haplotypes. Similarly, in HsInv0209, HsInv0278 or HsInv0397 there were few individuals or a limited number of SNPs in the inverted region to draw reliable conclusions, but a unique origin of the inversion could not be discarded according to existing evidence. In HsInv0278, there are only two close HapMap SNPs within the inverted region giving rise to three haplotypes shared between *Std* and *Inv*. However, in the 1000GP data, the analyzed *Inv* chromosomes correspond to the same HapMap haplotype and cluster together, which indicates that we are only observing part of the variation.

On the other hand, *Std* and *Inv* haplotypes of four inversions (HsInv0341, HsInv0344, HsInv0389, HsInv0393) were dispersed on the trees and networks (Figure 5C and 5D). This is consistent with the high number of shared SNPs found between arrangements for all these inversions and suggests that at least some of them have appeared recurrently several times. For example, in HsInv0341 there are three inverted chromosomes included in two highly divergent haplotype clusters of *Std* chromosomes (Figure 5C), suggesting that the inversion has occurred independently twice. Something similar could have happened in HsInv0393, in which we observed two main HapMap haplotypes that include mostly chromosomes of one orientation and a few of the other one, although in the 1000GP data *Inv* haplotypes clustered with *Std* ones are missed due to the lack of individuals. In HsInv0389, which was previously found to be recurrent across mammals [59], there are two highly divergent *Std* and *Inv* haplotype clusters, probably derived from an initial inversion event. Within each of these groups, a few haplotypes are found with the opposite conformation suggesting two additional inversion and re-inversion events (Figure 5D). Similar complex networks with divergent clusters of mixed *Std* and *Inv* haplotypes could be observed in HsInv0344, which are compatible with two or three inversions and re-inversions. Lastly, HsInv0241 and HsInv0403 are small inversions that present few HapMap SNPs within the inversion. For the 1000GP set, there were two different haplotypes shared between *Std* and *Inv* chromosomes. However, these two haplotypes are just defined by a small number of SNPs separated by 1–2 kb and could be explained either by inversion recurrence or a long gene conversion tract.

### Orientation of the inversions in other species

The iPCR technique is especially sensitive to the loss and generation of additional restriction sites, and given the low quality of most non-human primate reference genome sequences, it is not easy to transfer the assays to other species. However, we examined the orientation of the 17 human polymorphic inversions in non-human primates by iPCR. In most cases, the human iPCR assay could be applied to the other species, but for five regions we had to develop a completely new assay or modify the existing one (Table S1 and Table S6). Using DNAs from four chimpanzees and two gorillas (including a father-son pair in each species), we obtained reliable results from at least one breakpoint in one species for 16 inversions (Table 2 and Figure S3), and there was information from both breakpoints for 11 inversions in chimpanzees and five inversions in gorillas. Of those 16 inversions, nine (56.3%) were polymorphic in at least one of the non-human primate species, including HsInv0403 that was polymorphic in the three species analyzed (Table 2). In addition, HsInv0832 showed different orientations in chimpanzees and gorillas. Thus, this provides further support for the existence of several independent origins for these inversions.

To complement the above results, we checked the orientation of these regions in current primate genome sequences by dot plot and Blast. Chimpanzee genome orientation agreed perfectly with that obtained in the iPCR, whereas in gorilla there were several discrepancies that could be due to the quality of the current assembly and the difficulty of assembling regions flanked by IRs (Table 2). By combining the iPCR information with the position of the Denisovan, chimpanzee and rhesus sequences in the haplotype trees (Figure S2), we could estimate the ancestral orientation for 14 of the human polymorphic inversions (Table 2). In general, there was a good correspondence between the seemingly oldest arrangement and a higher frequency and increased levels of nucleotide variation in the CEU population. However, there were some interesting exceptions to be studied in more detail. For example, in HsInv0114 the *Inv* orientation was found in both outgroups and showed a >60% frequency in CEU populations, although both the haplotype tree and the nucleotide variation values suggest that the *Std* orientation might be ancestral. Similarly, in HsInv0278 the supposedly ancestral *Inv* orientation showed a lower frequency and less nucleotide diversity than *Std* chromosomes. Finally, for HsInv0340 the *Inv* orientation is missing in the CEU population, although it is ancestral according to the gorilla iPCR results.

### Functional effects of inversions

To investigate the possible functional consequences of the inversions we also analyzed their association with genes annotated in the HG18 and HG19 genomes (RefSeq and UCSC genes, http://genome.ucsc.edu), especially those genes located within the inverted sequence or spanning the predicted breakpoints (Table 2). In total, seven inversions do not contain genes in the inverted or breakpoint regions, and two just change the orientation of genes within the inversion. One of these (HsInv0389) inverts the *FLNA* and *EMD* genes and has been associated to a deletion causing Emery-Dreifuss muscular dystrophy [9], [10]. Here, we provide an easy method to identify the inversion carriers. In the other eight inversions, the predicted breakpoint regions overlap with described genes (Table 2), although the expected consequences on the genes are variable.

In three cases (HsInv0124, HsInv0344, and HsInv0393), the exonic sequences of genes located within the breakpoint region are 100% identical in the two SDs implicated in the inversion. Therefore, if there is an exchange between the SDs, the sequence of the mRNA should not be affected. In four inversions, the SDs contain entirely or in part two genes of the same family. These include two copies of *tRNA-Val* and *tRNA-Leu* in HsInv0278 and the *AQP12A* and *AQP12B* genes in HsInv0241, in which as before the exchange between them should not affect the genes. For example, HsInv0241 exchanges the first exon of *AQP12A* and *AQP12B*, where all the differences between the two copies are located, and apparently just alters the relative position of the genes. For HsInv0209, there are two inverted SDs of 7 kb and 94% average identity that include completely the *KRTAP5-10* and *KRTAP5-11* genes (85% identity). Due to the lack of *Inv* arrangement sequence data, the inversion breakpoints have not been refined within the duplications. However, the most likely location of the breakpoints is a 782 bp region with 99.9% identity between SDs, which is >2.4 kb away from the 3′ ends of both genes. Finally, for HsInv0396, almost the entire *PABPC1L2B* and *PABPC1L2A* genes are included within the possible breakpoints and contain three single nucleotide variants, although they do not produce an amino acid change and appear to be polymorphic between different copies of the same duplication. Thus, again the inversion will just change the relative orientation of the two gene copies by exchanging the last part of the 3′UTR, which is the most divergent between them.

The best example of a gene disruption caused by an inversion is that of *CCDC144B*, which spans BP1 of HsInv1051. This gene spreads over 87.8 kb, with several coding exons at both sides of the SD implicated in the inversion, and the inversion moves the two first exons 200 kb away from the rest. *CCDC144B* is part of a family with two other members, *CCDC144A* and *CCDC144C*, with >99.1% identity and very similar exon-intron structure. Nevertheless, whereas *CCDC144A* encodes a protein of 1365 amino acids, *CCDC144B* and *CCDC144C* have different frameshift changes that reduce their coding capacity to 725 and 647 amino acids, respectively. The possible function of these proteins is not clear and it has been considered that both genes could be pseudogenes, although the expression of *CCDC144B* is supported by multiple mRNAs and ESTs. Interestingly, the HsInv1051 inversion is not present in the CEU population and so far has been found in a single YRI family, which suggests that it has a relatively low frequency maybe related to its effect on the gene. When we looked in detail to the effect of the breakpoints of all the inversions in other mRNAs, spliced ESTs and ENCODE/GENCODE (version 14) gene annotations, there are several non-coding RNAs that could be affected. One clear case is that of HsInv0340, that truncates the putative *LINC00395* RNA supported by three spliced ESTs. However, further work is needed to explore the possible functionality of this RNA.

## Discussion

Information on human inversions has been typically scarce due to the technical difficulties in their experimental validation and genotyping. Here, we describe an optimized protocol to genotype a big fraction of inversions in a fast and high-throughput fashion. Although the iPCR method had been used before to study individual inversions [47], we have demonstrated that it can be scaled easily to a large number of inversions with high reproducibility, with just minor problems in a few of the assays. In addition, the elimination of intermediate purification steps allowed us to use much smaller amounts of DNA per sample (<100 ng in front of 1 µg [47]). Finally, through the optimization process we have shown that with appropriate high-molecular weight DNA, iPCR can be used for large DNA fragments of more than 64 kb, which is three times bigger than in previous studies [47]. This makes it possible to analyze inversions mediated by long IRs (up to ∼25 kb in our case), which are very difficult to genotype by other methods. Therefore, the iPCR fills a gap in the study of inversions and increases considerably the range of inversions that can be genotyped. Moreover, iPCR could also be useful in the analysis of other structural variants, like translocations, or complex genomic regions in which the exact organization is not clear.

One of the main limitations of the iPCR is the availability of restriction sites in the regions of interest that generate fragments of a size that can be efficiently recircularized (<70 kb). In the case of inversions mediated by IRs, this means having restriction sites exclusively outside the repeats and within the inverted region, which could be quite difficult when the inversion is small or has large SDs. In particular, we have seen that the iPCR protocol works very consistently for restriction enzymes with staggered ends, but the efficiency decreases considerably for blunt-end enzymes. Thus, in order to increase the number of inversions that can be analyzed, future improvements of the technique should be directed to increase the efficiency of the recircularization, especially for big fragments and fragments with blunt ends, and the possibility of making directed cuts in the regions of interest. In addition, the iPCR design relies on a good sequence assembly, and compared with other genotyping techniques, it is more sensitive to changes on the overall organization of the region. For example, the iPCR amplification could be affected by indels or structural variants, which could create new restriction sites or modify the size of the resulting fragments, or other types of restriction site polymorphisms. Therefore, for complex and highly-variable regions it might be difficult to interpret the results, and it is important to check the consistency between the two breakpoints. We have observed differences between the results of the two breakpoints in a few individuals for three of the 17 validated inversions, although in all cases we were able to deduce that an allele was missing in one of the assays.

Thanks to the iPCR, we have confirmed the organization of a complex region that was incorrectly assembled in the human genome and invalidated two inversion predictions caused by sequence differences between SDs. Moreover, we have validated 17 polymorphic inversions, showed that all of them behave as normal genetic variants, and obtained a first estimate of their frequency in the CEU population. This represents one of the biggest studies of human inversions both in the number of inversions and individuals analyzed. Only one of these inversions, HsInv0389, had been previously detected at a frequency of 18% in a sample of 108 chromosomes from individuals of European descent [9], which is very similar to the frequency we obtained (17.6%). Interestingly, two inversions (HsInv0832 and HsInv1051) were not found in CEU individuals and their frequency could differ between populations, as it has been shown previously by SNP inference for the inversions in 17q21.31 [14] or in 8p23 [21]. Future studies of more individuals from other populations would provide a clearer picture of the worldwide distribution of these inversions.

Traditionally, especially from studies in *Drosophila*, it has been assumed that inversions have a unique origin and are monophyletic [60]. Consistent with this, our analysis of the association between the inversion and SNP genotypes has shown that several of them have probably a unique origin and are labeled by tag SNPs, at least in the CEU sample. However, for four other inversions the same region appears to have gone through several inversion and re-inversion events in the human lineage. These inversions are characterized by: i) the absence of fixed SNPs and a very high amount of shared SNPs between arrangements, reaching in some case the totality of the SNPs present in the *Inv* arrangement (Figure 4); and ii) *Std* and *Inv* haplotypes spread on the networks and trees with sometimes more than one shared haplotype (Figure 5). There are two additional inversions that show this same pattern, but the number of SNPs affected is too small to reach any conclusion. To make sure that these results are not affected by genotyping errors, all the individuals indicating inversion recurrence were genotyped at least twice for both breakpoints, especially when recurrence was based only on a few individuals, and their identity was confirmed by microsatellites. In addition, for the four inversions showing the highest number of shared SNPs in the 1000GP data, ∼20% of them were re-sequenced and confirmed. Finally, it is worth mentioning that, in most cases, inversion recurrence was based on independent SNP data from HapMap and 1000GP, and it was supported by both simple genotype data and haplotype (phased) data. In particular, the HapMap haplotypes were inferred using trio information, which minimizes phase errors, and were consistent with those obtained for the 1000GP data.

Assuming that the occurrence of identical independent mutations across several positions is very unlikely, the only other mechanism besides inversion recurrence that could explain the level of genetic flux observed between arrangements is recombination (either gene conversion or double crossovers). Current recombination estimate in humans is ∼23 crossovers per cell (approximately one per chromosome arm) [61], which given the size of the inversions and the phenomenon of crossover interference [62] makes the possibility of double crossovers within the inverted region very small. In addition, gene conversion tracts in mammals are usually short, extending for only a few hundred base pairs, and are rarely longer than 1 kb [63] (although some studies suggest the existence of gene conversion events up to 22 or 53 kb; see [61], [64]). Similarly, a recent high-resolution recombination map in *Drosophila melanogaster* found an average gene conversion tract length of 518 bp, with a 95% confidence interval in most chromosomes of less than 800 bp [65]. In fact, there is evidence of small gene conversion tracts that explain the limited number of shared SNPs between arrangements in some of the monophyletic inversions in this and other studies [21], [64]. However, the existence of many shared SNPs and identical haplotype blocks in *Std* and *Inv* chromosomes along the whole inverted region (Figure 4), with sizes between 5.6 and 37.6 kb, contrasts with the pattern observed in inversions mediated by non-homologous mechanisms (David Vicente and Mario Cáceres, unpublished data) and strongly suggests the recurrence of the inversions. Unfortunately, it is not possible to check if the breakpoints of the diverse inversion events are different since they all occur in regions of high sequence identity.

Moreover, when a few chimpanzees and gorillas were analyzed, we found that nine inversions were polymorphic in at least one species and another one showed different orientations between them, suggesting that they have occurred independently in these lineages. An alternative possibility is that these inversions were shared polymorphisms from the common ancestor. However, preliminary estimates of the age of the inversions in humans indicate that they are less than 350,000 years old, and the more than 6 million years of divergence between these three species makes this explanation unlikely. Together with the mammal recurrent inversion HsInv0389 [59], human inversions with multiple origins in primates include the six inversions showing a high number of shared SNPs between arrangements in humans, plus five that seemed unique or were not polymorphic in the CEU population. Therefore, this brings the total number of inversions showing signs of recurrence within humans or between different species to 11 of the 16 that could be analyzed (69%).

Previously, recurrence of SVs was known for those causing genomic disorders, like the inversion causing hemophilia [66]. For polymorphic inversions found in natural populations, the recurrence of inversions mediated by NAHR between SDs had already been postulated by comparison of different lineages in mammals [59] or primates [21], [67], and there was some evidence that it could occur in humans as well [12], [68]. In addition, experimentally it was suggested that some inversions could appear repeatedly in human cells [69], [70], although these results should be confirmed with independent techniques. Nevertheless, inversion recurrence had never been demonstrated to the extent shown here, with in some cases signs of several inversion and re-inversion events of the same region both within and between species. Interestingly, the possible recurrent inversions are a representative sample of all the analyzed inversions in terms of size and breakpoint features. Thus, high levels of recurrence could be a characteristic of inversions mediated by large IRs, and it would be interesting to see if even more inversions will show a polyphyletic origin when additional human populations or individuals of other species are analyzed.

The observed high incidence of inversion recurrence has two important consequences. First, for many of the inversions mediated by IRs, which are probably a large fraction, genotypes could not be inferred from SNP information but must be resolved experimentally. Second, genome-wide association studies based on SNP genotyping would miss the phenotypic effects of most inversions. In general, according to our bioinformatic predictions, the functional consequences of the studied inversions on the flanking genes is expected to be small, with 41% in which there is not any gene around the breakpoints and many others that do not affect the gene mRNA. However, there are two inversions that disrupt a possible coding gene and a long non-coding RNA, and additional experiments are needed to analyze the effects on the expression of these genes across multiple tissues. Also, it would be interesting to check other possible effects of the inversions on more remote genes. The availability of reliable assays to genotype inversions would allow us to make associations of inversions and gene-expression levels. In addition, it makes possible to genotype groups of human samples with particular phenotypes. Therefore, this study not only gives us a better idea of the distribution and evolutionary history of inversions in human populations, but opens the possibility to determine their functional consequences in the near future.

## Materials and Methods

### Human samples and DNA extraction

We used a total of 83 human samples from the HapMap project [52] (with the exception of NA15510), including 70 CEPH individuals with ancestry from northern and western Europe (CEU), 10 Yoruban individuals (YRI), and two individuals from China (CHB) and Japan (JPT) (Table S3). The 70 CEU samples corresponded to 46 independent individuals, with 12 parent-child pairs, and 12 complete trios. Genomic DNA was obtained from Epstein-Barr virus-transformed B-lymphoblastoid cell lines of each individual (Coriell Cell Repository, Camden, NJ, USA). DNA was extracted from a 40-ml cell culture grown according to the recommended procedures using standard phenol-chloroform extraction protocol with modifications to obtain high molecular weight DNA [71]. Identity of all the DNAs extracted was confirmed using the MSK microsatellite kit (Coriell Cell Repository, Camden, NJ, USA). In addition, DNA samples from four chimpanzees and two gorillas were also used. DNAs from both gorillas and chimpanzee N457/03 were isolated from frontal cortex brain tissue obtained from the Banc de Teixits Animals de Catalunya (BTAC, Bellaterra, Barcelona, Spain). The remaining chimpanzee DNAs were extracted from Epstein-Barr virus-transformed B-lymphoblastoid cell lines generated from blood of three individuals from the Barcelona Zoo. All procedures involving the use of human and non-human primate samples were approved by the Animal and Human Experimentation Ethics Committee (CEEAH) of the Universitat Autònoma de Barcelona.

### Inversion selection and breakpoint definition

Inversions to test were selected from the analysis of the fosmid PEM data from Kidd et al. [23] using our own inversion predicting algorithm GRIAL, as described in the InvFEST database [54]. To identify and refine the IRs possibly involved in the rearrangement, the sequence of the predicted inversion in the HG18 genome version was self-aligned with BLAST at the NCBI website [72]. In addition, whenever possible, the position of the breakpoint regions was delimited by multiple sequence alignment of the IRs from the *Inv* and *Std* arrangement using available human genome and whole fosmid sequences [54]. The breakpoint boundaries were defined by three or more contiguous paralogous sequence variants (PSVs) that get exchanged between the two copies of the IRs in the individuals with the inversion.

When necessary, re-mapping of the fosmid paired-end reads to HG18, HG19 or individual genome patches was carried out using the SMALT v.0.6.1 program (http://www.sanger.ac.uk/resources/software/smalt/). Reads were mapped independently and for each one we kept track of the top 10 mappings with >90% identity, Smith-Waterman scores above 300, and differing <5% from the hit with highest score. Fosmids with two mapped reads were classified into three categories: concordant, discordant in orientation, and ambiguous. Concordant pairs map with the expected orientation (+/−). Discordant pairs are candidates to target an inversion breakpoint and one of the reads shows the opposite orientation (+/+ or −/−). We consider a pair to be ambiguous when one or both reads map to a copy of a highly-identical IR region (defined using MEGABLAST as sequences spanning ≥1 kb and ≥97% identity) and it has both concordant and discordant alternative mappings.

### Inverse PCR (iPCR) design and optimization

For the iPCR assays, we selected restriction enzymes with target sites outside the IRs and at each side of the inversion breakpoints that create fragments between 2.3–73 kb using NEBcutter [73] (Table 1 and Table S1). Then, for each inversion, at least four primers (Table S6) pointing outwards close to the restriction site at each side of the fragment were designed with Primer3plus [74] to amplify the AB and CD fragments (*Std* orientation) and the AC and BD fragments (*Inv* orientation) (Figure 1). Both primers and restriction sites were checked against NCBI dbSNP Build 137 to avoid differences in the assay due to genetic variation between individuals. In addition, to ensure PCR specificity, no more than one primer in each pair was located in repetitive regions and no reliable amplification was predicted in the human genome by the NCBI Primer-BLAST tool [72].

To optimize the iPCR process, 2600 ng of NA18517 genomic DNA were digested overnight in a 130-µl volume reaction containing 40 U of *BamHI* (Roche) or *SwaI* (New England Biolabs) at 37°C and 25°C, respectively, followed by heat inactivation of the enzymes at 65°C for 15 min. Digested DNA was self-ligated for 3.5 hours at 25°C in six reactions (400 ng each) using 400 U of T4 DNA ligase (New England Biolabs) in different final volumes to obtain DNA concentrations of 10, 5, 2.5, 1.25, 0.62 and 0.31 ng/µl. The ligase was inactivated at 65°C for 10 min, and the final volume of all reactions was standardized to 1280 µl with 1× ligation buffer. Then, DNA was purified once with phenol-chloroform and chloroform-isoamyl alcohol, precipitated with 2.5 volumes of ethanol and 0.1 volumes of sodium acetate 3M pH 5.2, and resuspended in 50 µl of water. To assay the effect of different chemical reagents, the process was the same, with the exception that 640 µl ligation reactions containing 0.62 ng/µl of DNA were carried out with 2% polyethylene glycol (PEG), 10% glycerol, 1 µg/ml glycogen or 1 µg/ml bovine serum albumin (BSA). Two ligation reactions without any added reagent were used as control.

### Quantitative real-time PCR

Self-ligation efficiency at different DNA concentrations or chemical reagents tested during iPCR optimization was compared by quantitative real-time PCR using the iTaq SYBR Green Supermix with Rox (BioRad) in an ABI Prism 7500 Real-Time PCR System (Applied Biosystems). NA18507 circularized DNA in each ligation was amplified with iPCR primers specific for the two inversion arrangements, using the *CLIP2* gene as a reference to control for DNA differences (Table S6). Amplification conditions were an initial denaturation step of 2 min 45 s at 95°C, followed by 40 cycles of 95°C for 15 s and 60°C for 50 s, and a final dissociation step. For each condition, PCR amplifications were done in triplicate from approximately 13 ng of purified DNA. Real-time PCR results were analyzed using the Sequence Detector and Dissociation Curve programs (Applied Biosystems). Relative ratio quantification was calculated by the Pfaffl method [75] using the Ct values and efficiency of each primer obtained from the standard curve.

### High-throughput inversion genotyping by iPCR

For high-throughput iPCR, reactions were performed in a 96-well plate with slightly different conditions for fragments with staggered and blunt ends. Typically, 100–150 ng of genomic DNA were digested under conditions recommended by the manufacturer overnight in a 25 µl reaction with 3 U of staggered-end restriction enzymes (*EcoRI, HindIII, SacI, BamHI*, Roche; *NsiI, BglII*, New England Biolabs). Alternatively, 500 ng of genomic DNA were digested overnight in a 25 µl reaction with 10 U of the blunt-end enzyme *SwaI* (New England Biolabs). Enzymes were inactivated at 65°C for 15–20 min (for *BglII* 20 min at 85°C). Self-ligation of 20 µl of digested DNA was performed for 3 hours at 25°C in a total volume of 175 µl with 120 U of T4 DNA ligase (New England Biolabs). For blunt ends, self-ligation was done in 100 µl with 1 µg/ml of glycogen and 400 U of T4 DNA ligase (New England Biolabs). Ligation reactions were inactivated for 10 min at 65°C.

Circular DNA molecules were amplified directly without any further purification in 25 µl PCR reactions with 10 µl (∼5–7 ng and 40 ng of DNA for staggered- and blunt-ends, respectively) of the digestion and ligation mix (after vigorous vortexing 20–30 s to 3500 rpm), 1.5 U of Taq DNA polymerase (Biotherm), 0.4 µM of each primer (for multiplex PCR, 0.8 µM common primer and 0.4 µM unique primers), 0.2 mM dNTPs, and 1× Taq DNA polymerase buffer without MgCl_2_. Amplification was carried out by 5 min at 95–98°C, 33–35 cycles at 95°C for 30 s, 55–61°C for 30 s, and 72°C for 30 s, and a final extension at 72°C for 5 min. PCR products were analyzed by electrophoresis on ethidium bromide-stained 1.5–2% agarose gels. It is important to note that other Taq DNA polymerases failed to amplify the unpurified ligation products, probably due to buffer incompatibilities.

### Population genetics and nucleotide variation analysis

Inversion allele frequency, heterozygosity, and Hardy-Weinberg (HW) equilibrium were calculated for the CEU population sample considering only unrelated individuals with Arlequin v3.1 [76]. For chr. X inversions, HW equilibrium and heterorozygosity were calculated from female genotypes. To explore the nucleotide variation associated to the inversions, SNP data in a region from −10 kb to +10 kb from the IRs (excluding all SNPs within the IRs) were retrieved from HapMap [52] and 1000GP phase 1 [53]. These two databases were complementary since for the 1000GP fewer CEU individuals were available than for HapMap, although with more polymorphisms. Given the low sequence coverage of the 1000GP data, we performed two separate analyses with all or with only the most reliable SNP genotypes. To obtain a high quality 1000GP genotype set, we compared the likelihood of the given and the next most likely genotype, and discarded the genotypes in which it was not at least 10 times larger. Pairwise LD between polymorphisms was quantified by the *r^2^* statistic using Haploview v4.1 [77]. To avoid phasing errors, shared polymorphisms between *Inv* and *Std* chromosomes from genotype data were conservatively estimated based on the presence of polymorphic SNPs in *Std/Std* and *Inv/Inv* homozygotes or *Std/Inv* heterozygotes homozygous for both alleles of a SNP or for an allele polymorphic in one orientation and not in the other one.

Inference of the haplotype phase from HapMap and the 1000GP SNP data was carried out with PHASE v2.1 [57], [58] adding the inversion genotypes at the locations of the two breakpoints and using the available trio information when possible. We considered the inferred *Std* and *Inv* haplotypes as two sub-populations and differences between them were evaluated by means of Fst values with Arlequin v3.1. To investigate the relationships between haplotypes, we generated Median-Joining networks with Network 4.611 [78]. Neighbor-Joining trees were built with the Phylip v3.69 package [79] using the available alignments from the chimpanzee and rhesus genomes (Ensembl Release 66) or Denisova hominin genome [80] as outgroups. Measures of nucleotide diversity (π) were calculated with DnaSP version 5.10.1 [81].

## Supporting Information

Figure S1 Schematic representation of the HsInv0306 and HsInv0710 inversion prediction region in HG18 (bottom) and GL949743.1 patch (top). Collinear blocks between the two sequences are depicted on a purple background. The new inverted duplication in the patch is indicated by solid lines and regions with more than 97% identity between the duplications are labeled as u, v, w, x, y, and z (SD1) and u′, v′, w′, x′, y′, and z′ (SD2). An additional 15.8 kb region that is deleted in SD2 between z′ and y′ in some individuals is represented on top of the diagram. Unique discordant-in-orientation and concordant paired reads from the remapping of the fosmid data in the patch are linked by dashed lines, with reads mapping to the negative strand as yellow boxes (concordant paired reads, below) and reads mapping to the positive strand as red boxes (discordant-in-orientation paired reads, above). HsInv0306 inversion corresponds to original GRIAL predictions HsInv0306 and HsInv0312, whereas HsInv0710 inversion corresponds to original GRIAL predictions HsInv0710 and HsInv0311 [54]. In the remapping analysis, inversion HsInv0306 is supported by 19 unambiguously discordant fosmid paired-end reads, but this mapping profile is compatible with the polymorphic deletion of a genomic fragment between duplications z′ and y′, which causes that the end reads that should map concordantly within this region map within SD1 instead. The existence of this polymorphic indel was confirmed by the analysis of the mapping distance of the fosmid ends across this region (with only three individuals having fosmids consistent with the deleted form of SD2) and additional available human BAC sequences (AC245519 and AC245187, both including the SD2 extra sequence). According to this scenario, the presence of HsInv0306 and HsInv0710 is not supported anymore on the basis of the paired-end mapping data.
(TIF)

Figure S2 Median-Joining networks and Neighbor-Joining trees in CEU individuals for the 14 polymorphic inversions using the 1000 Genomes Project (A and C) and HapMap (B) SNP data. Haplotypes having the standard or inverted orientation are indicated in yellow and blue, respectively. In networks (A and B), circles represent the different haplotypes found for the region of the inversion and circle sizes are proportional to the frequency of each haplotype. Nucleotide changes between haplotypes are indicated as red numbers and red nodes correspond to hypothetical haplotypes. In trees (C), all sequences analyzed are represented in a different branch and black labels indicate the Denisovan sequences used as an outgroup. Bootstrap values >50 are shown based on 100 replications. Note that the two Denisovan sequences are not phased and do not represent real haplotypes. Alignments with chimpanzee (*Pan troglodytes*) and rhesus macaque (*Macaca mulatta*) have similar tree topology and location of the outgroup branch, although for many inversions the length of the sequence compared is much smaller. Trees were depicted with FigTree (http://tree.bio.ed.ac.uk/software/figtree/).
(PDF)

Figure S3 iPCR results for three human polymorphic inversions in non-human primate species. HsInv0393 shows both orientations in chimpanzees (CD and AC), but gorillas are inverted (AC). HsInv0403 shows both orientations (CD and BD) and is polymorphic in humans, chimpanzees and gorillas. HsInv0347 is polymorphic in gorillas (AB and BD), but the four chimpanzees are *Std* homozygotes (AB). Human samples are not included in HsInv0347 iPCR because a primer in region A specific for non-human primates was used.
(TIF)

Table S1 Summary information on the design of iPCR experiments to validate 21 inversion predictions in the human genome.
(XLSX)

Table S2 Consistency between the genotypes obtained by iPCR and the fosmid paired-end mapping (PEM) results in nine human individuals for 19 inversion predictions.
(XLSX)

Table S3 iPCR genotypes for 17 validated inversions in 83 human samples from CEU, YRI, CHB and JPT populations, four chimpanzees and two gorillas.
(XLSX)

Table S4 Tag SNPs in perfect linkage disequilibrium (*r* ^2^ = 1) with four human polymorphic inversions using data from HapMap and the 1000 Genomes Project (1000GP).
(XLSX)

Table S5 Nucleotide variation data from the 1000 Genomes Project (1000GP) for 14 polymorphic inversions with >2 inverted chromosomes in the CEU population after filtering the SNP genotypes based on the genotype likelihoods.
(XLSX)

Table S6 Primers and primer sequences used in the analysis of inversions.
(XLSX)
