# Supplementary figures and images for: Validation and Genotyping of Multiple Human Polymorphic Inversions Mediated by Inverted Repeats Reveals a High Degree of Recurrence
Source: PLoS Genet. 2014 Mar 20;10(3):e1004208. doi: 10.1371/journal.pgen.1004208 (PMC3961182; doi:10.1371/journal.pgen.1004208)

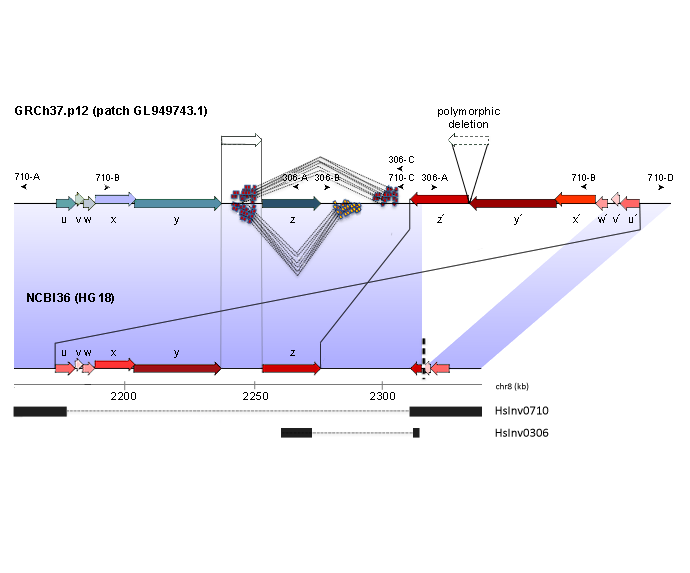

Supplement: Figure S1 — Schematic representation of the HsInv0306 and HsInv0710 inversion prediction region in HG18 (bottom) and GL949743.1 patch (top). Collinear blocks between the two sequences are depicted on a purple background. The new inverted duplication in the patch is indicated by solid lines and regions with more than 97% identity between the duplications are labeled as u, v, w, x, y, and z (SD1) and u′, v′, w′, x′, y′, and z′ (SD2). An additional 15.8 kb region that is deleted in SD2 between z′ and y′ in some individuals is represented on top of the diagram. Unique discordant-in-orientation and concordant paired reads from the remapping of the fosmid data in the patch are linked by dashed lines, with reads mapping to the negative strand as yellow boxes (concordant paired reads, below) and reads mapping to the positive strand as red boxes (discordant-in-orientation paired reads, above). HsInv0306 inversion corresponds to original GRIAL predictions HsInv0306 and HsInv0312, whereas HsInv0710 inversion corresponds to original GRIAL predictions HsInv0710 and HsInv0311 [54]. In the remapping analysis, inversion HsInv0306 is supported by 19 unambiguously discordant fosmid paired-end reads, but this mapping profile is compatible with the polymorphic deletion of a genomic fragment between duplications z′ and y′, which causes that the end reads that should map concordantly within this region map within SD1 instead. The existence of this polymorphic indel was confirmed by the analysis of the mapping distance of the fosmid ends across this region (with only three individuals having fosmids consistent with the deleted form of SD2) and additional available human BAC sequences (AC245519 and AC245187, both including the SD2 extra sequence). According to this scenario, the presence of HsInv0306 and HsInv0710 is not supported anymore on the basis of the paired-end mapping data. (TIF) [file pgen.1004208.s001.tif]

HsInv0114

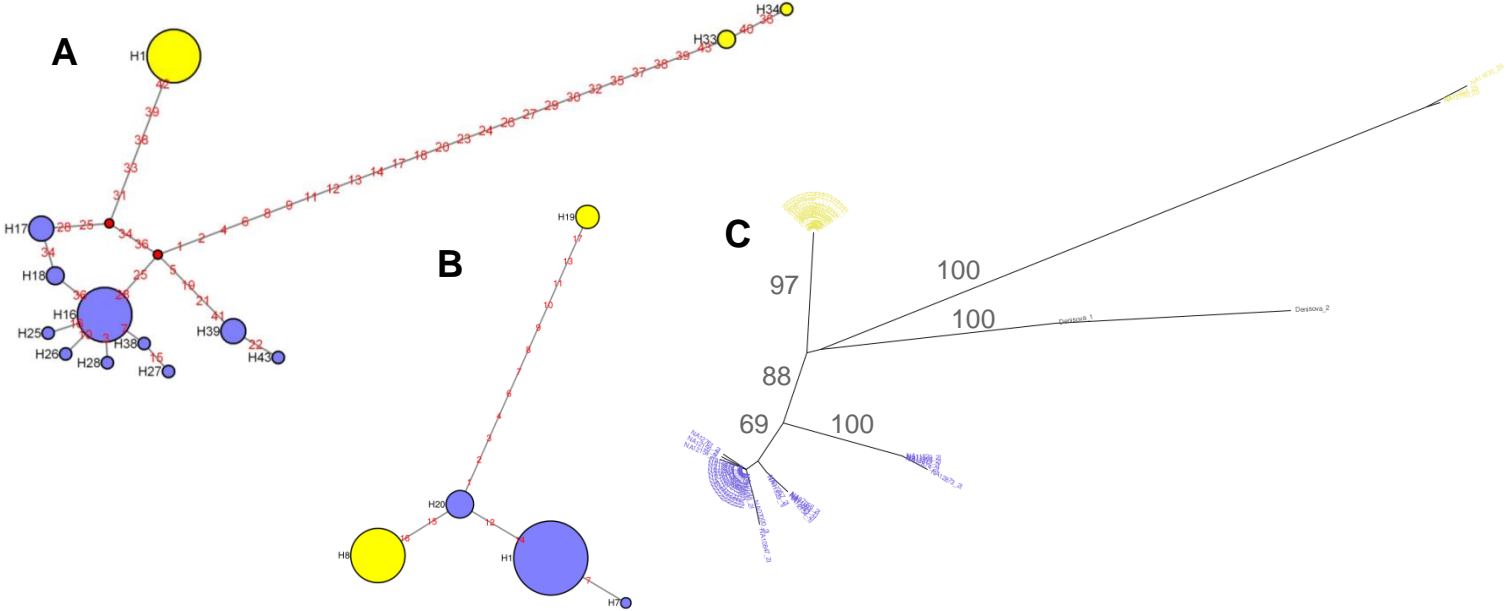

HsInv0124

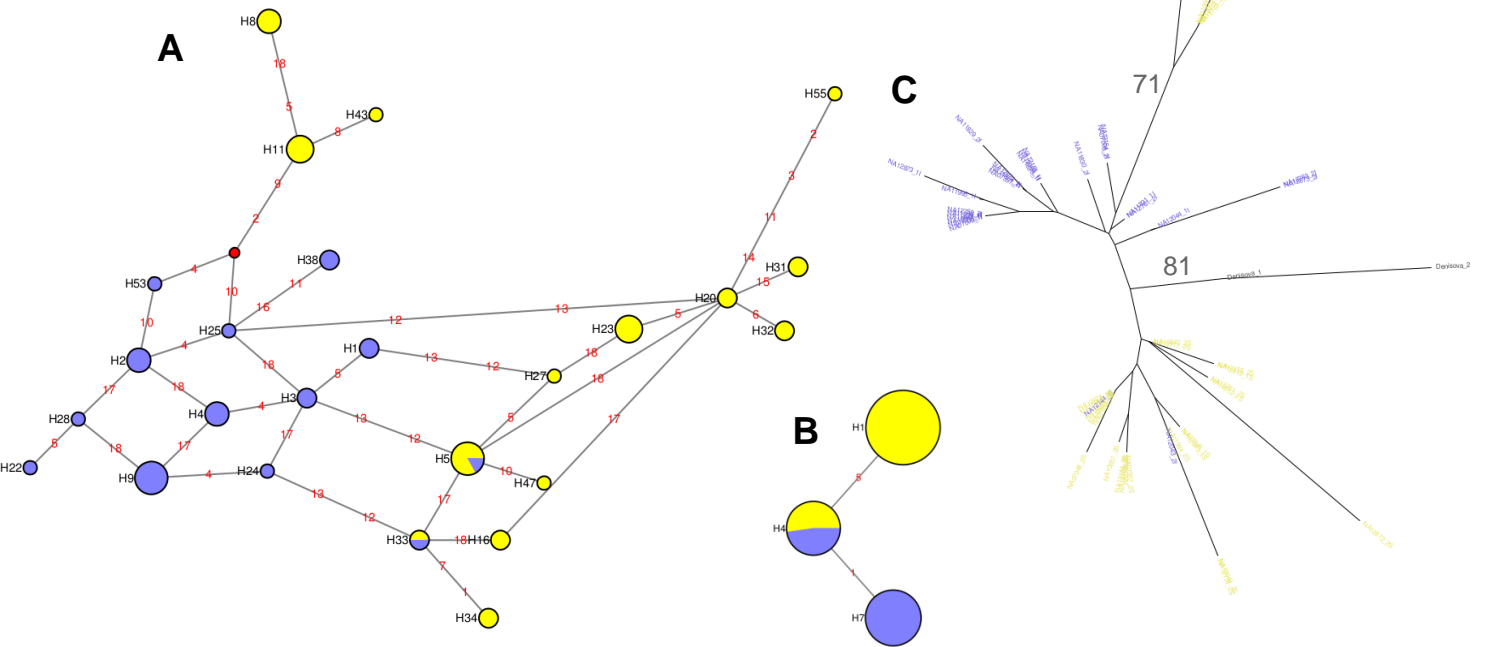

HsInv0209

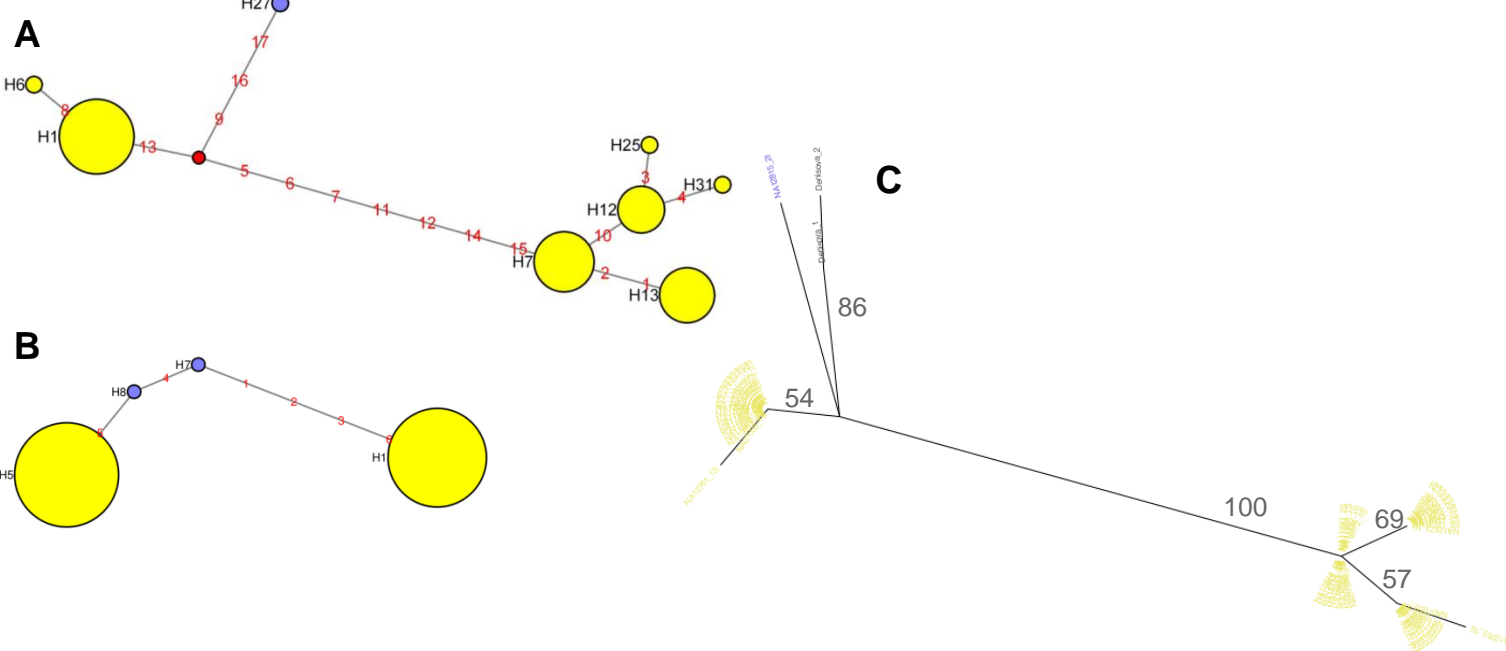

## HsInv0241

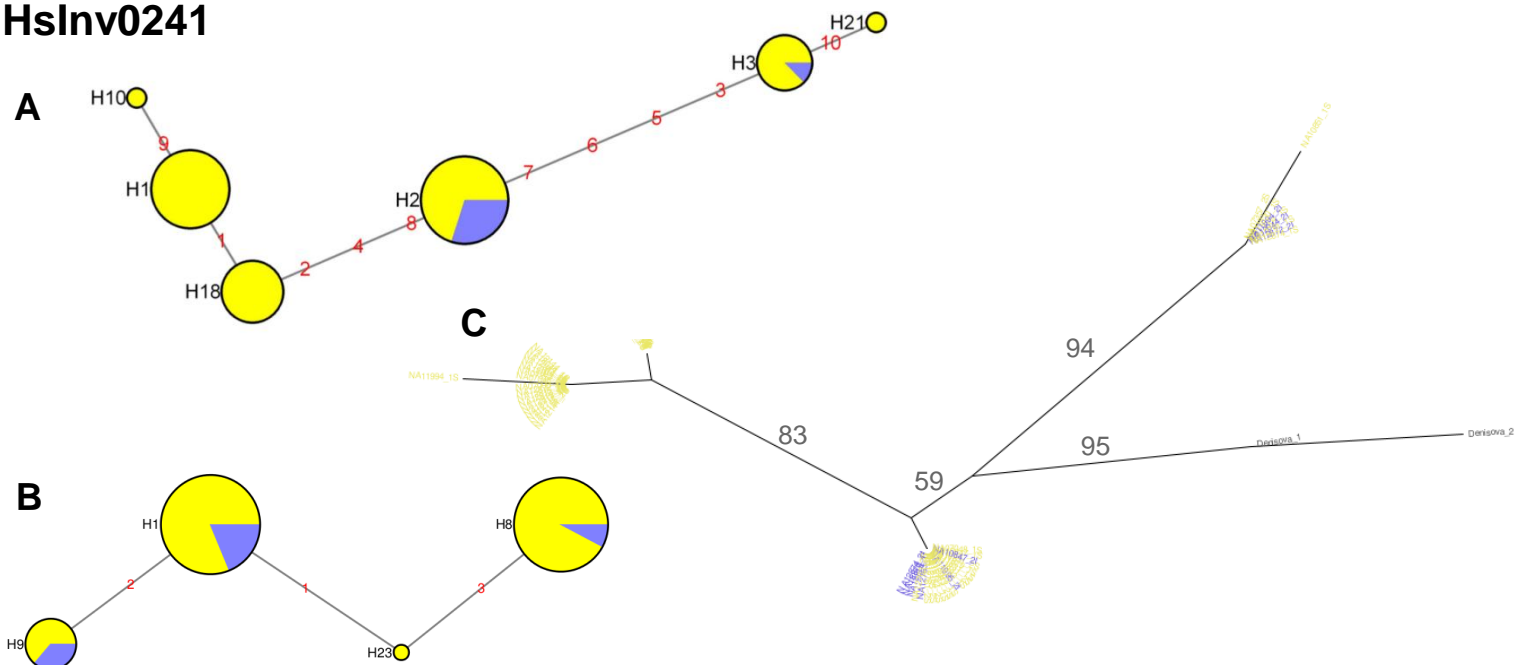

# HsInv0278

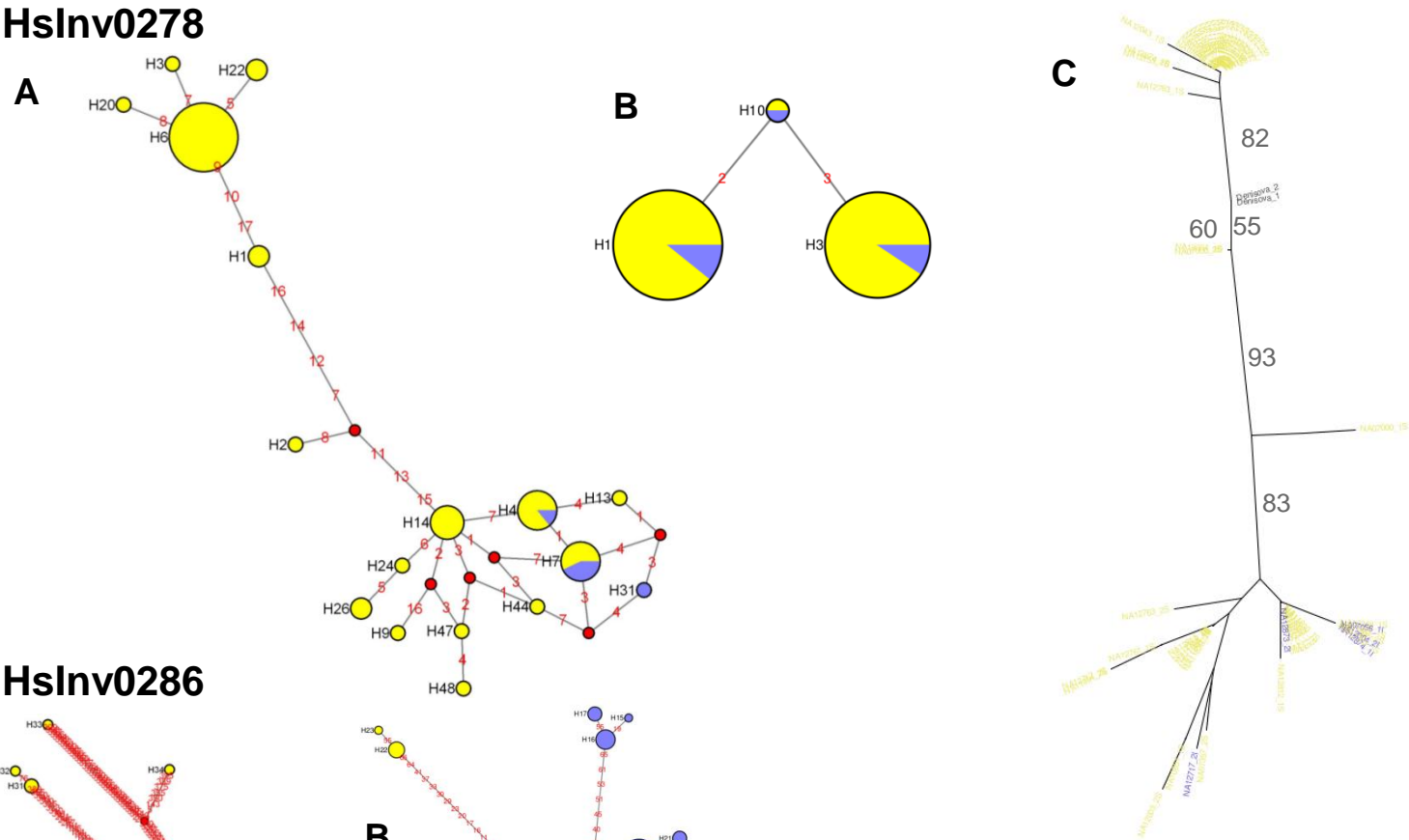

# HsInv0286

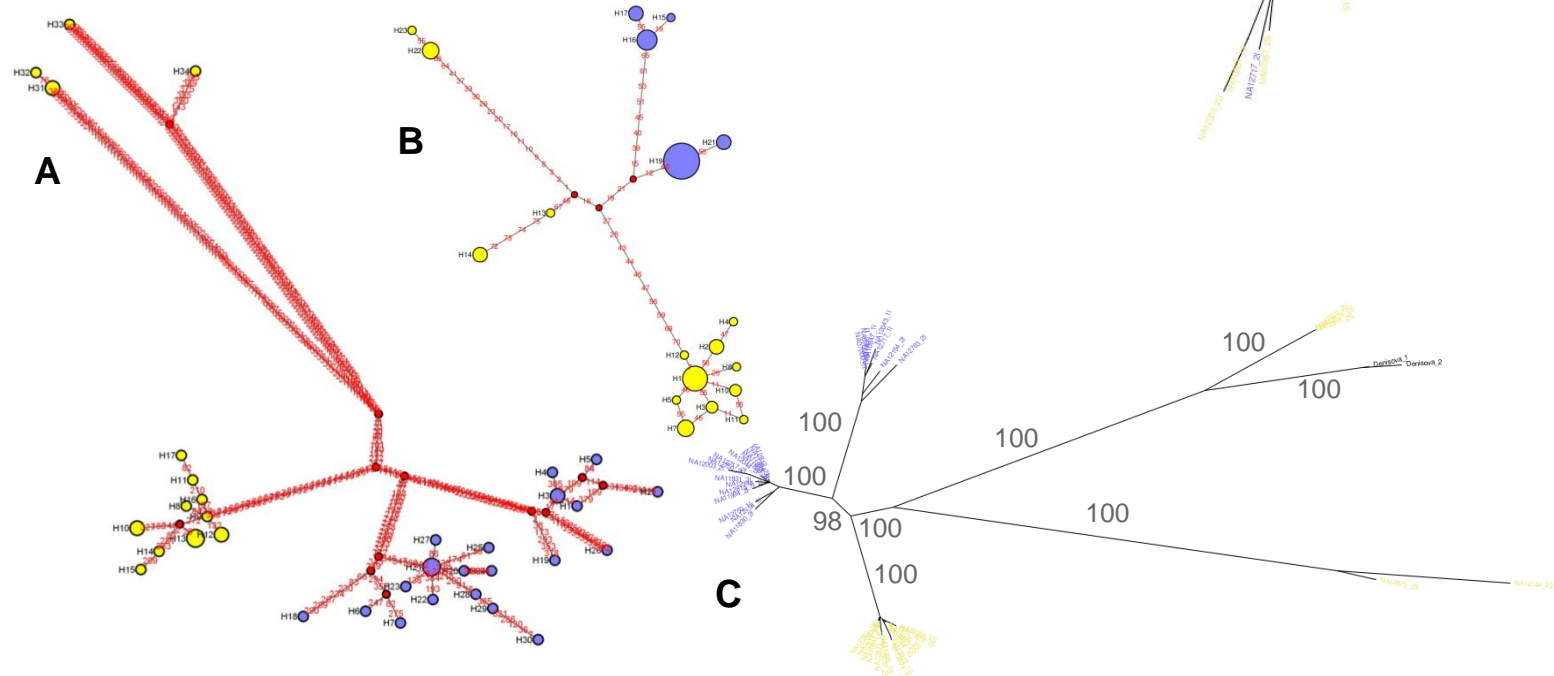

HsInv0341

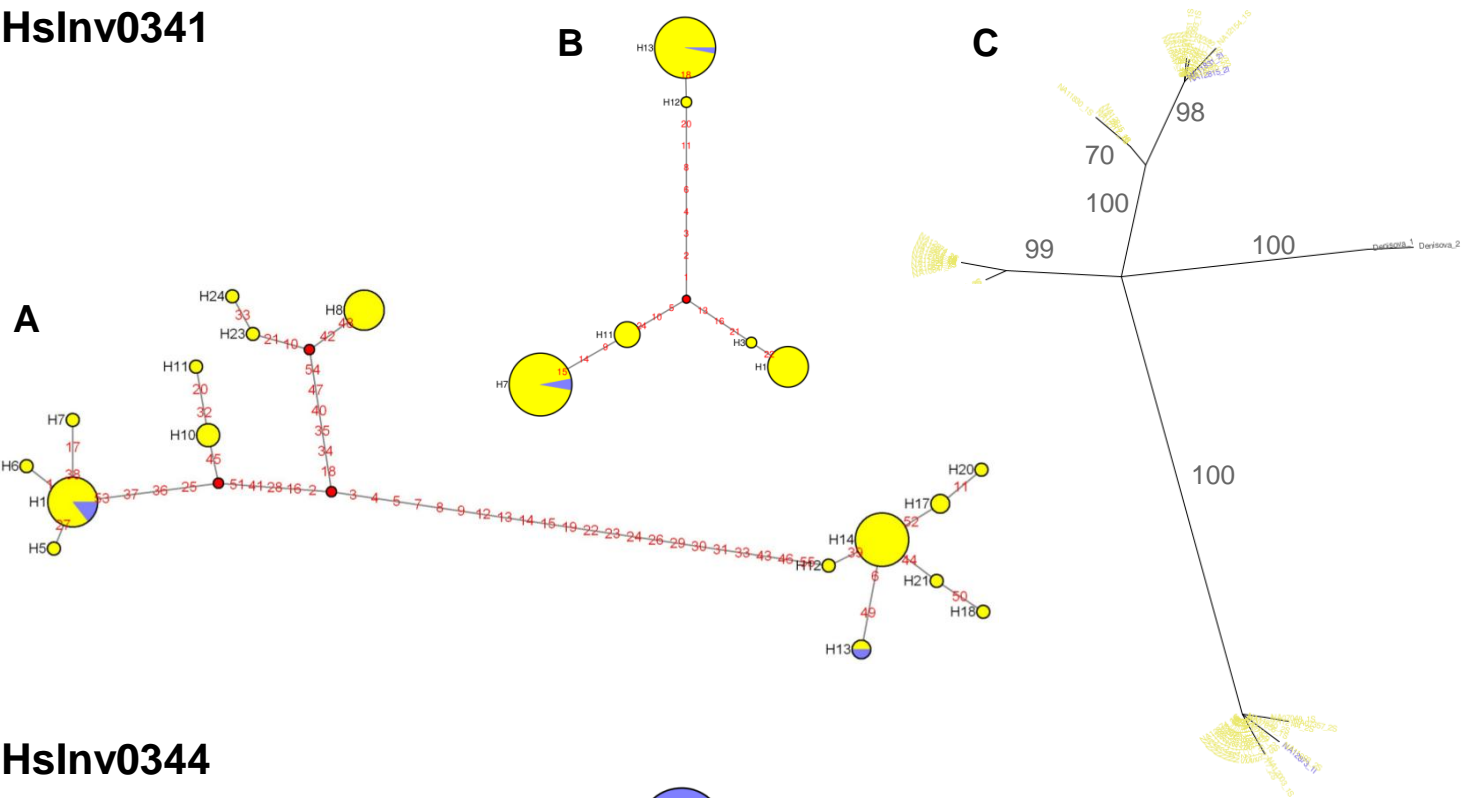

HsInv0344

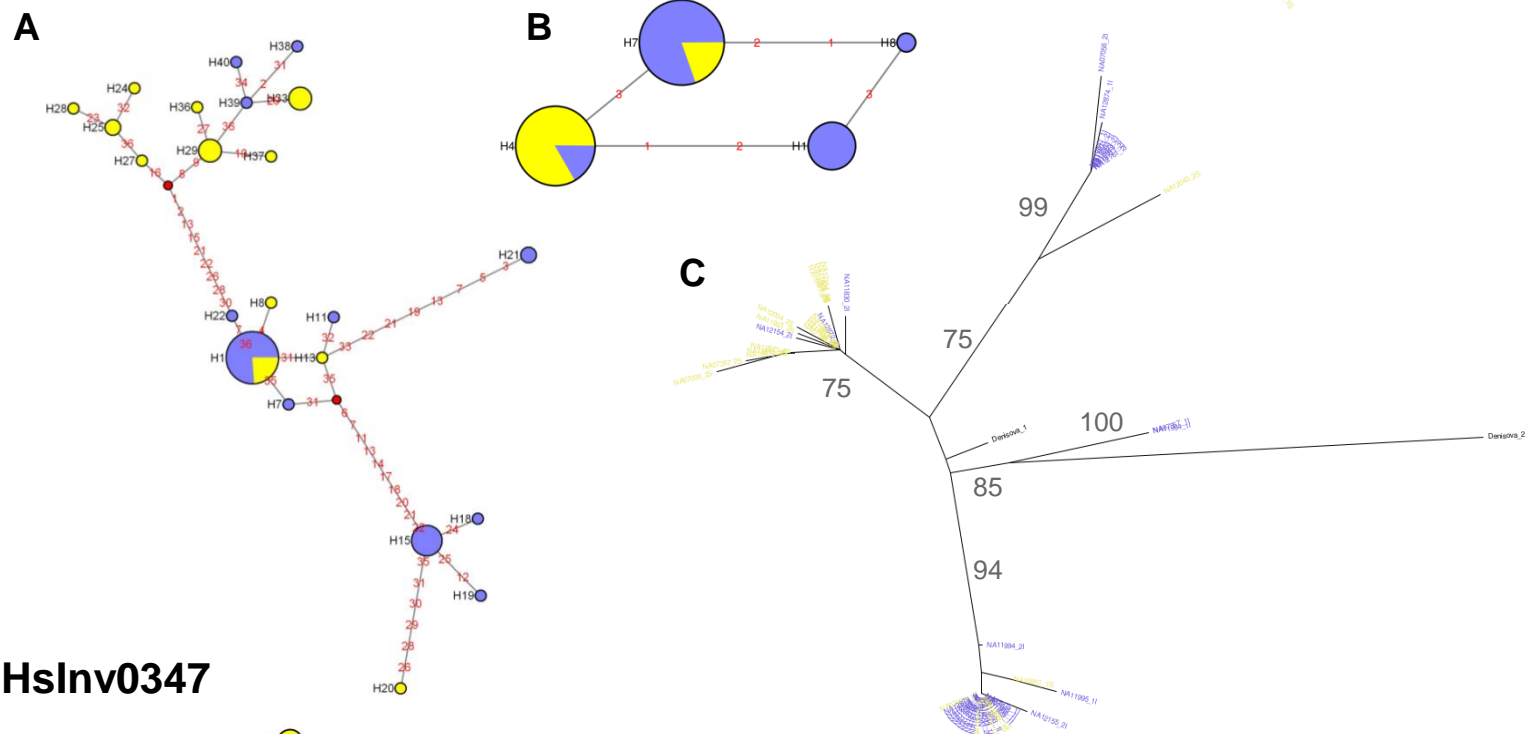

HsInv0347

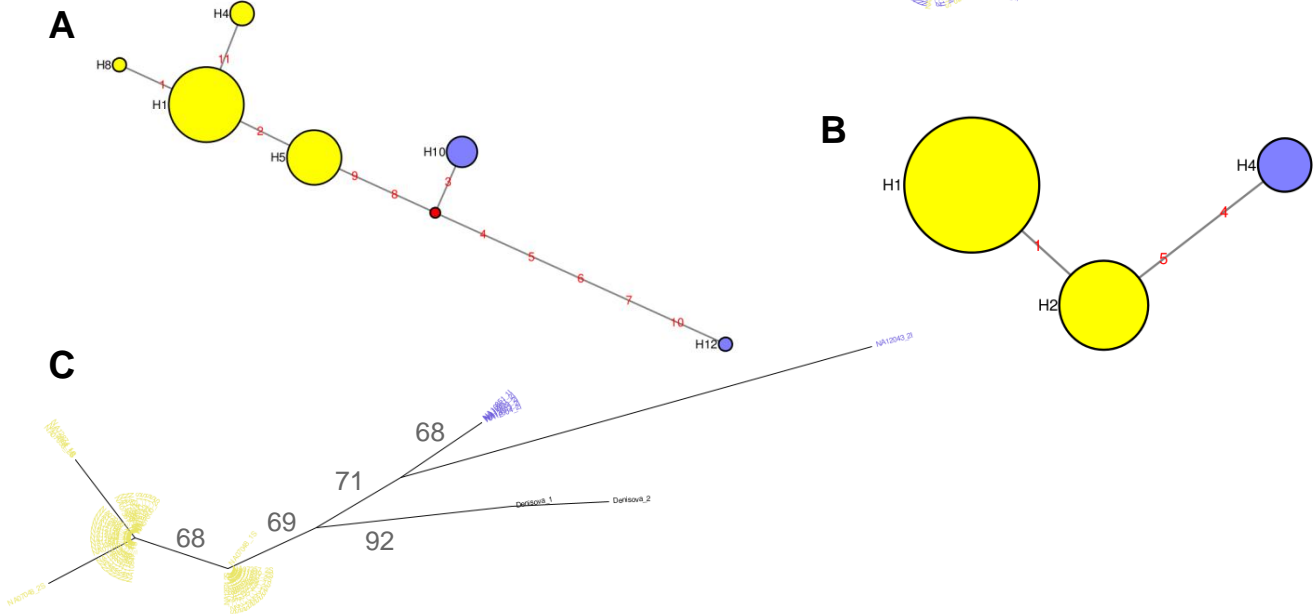

**HsInv0389**

**A**

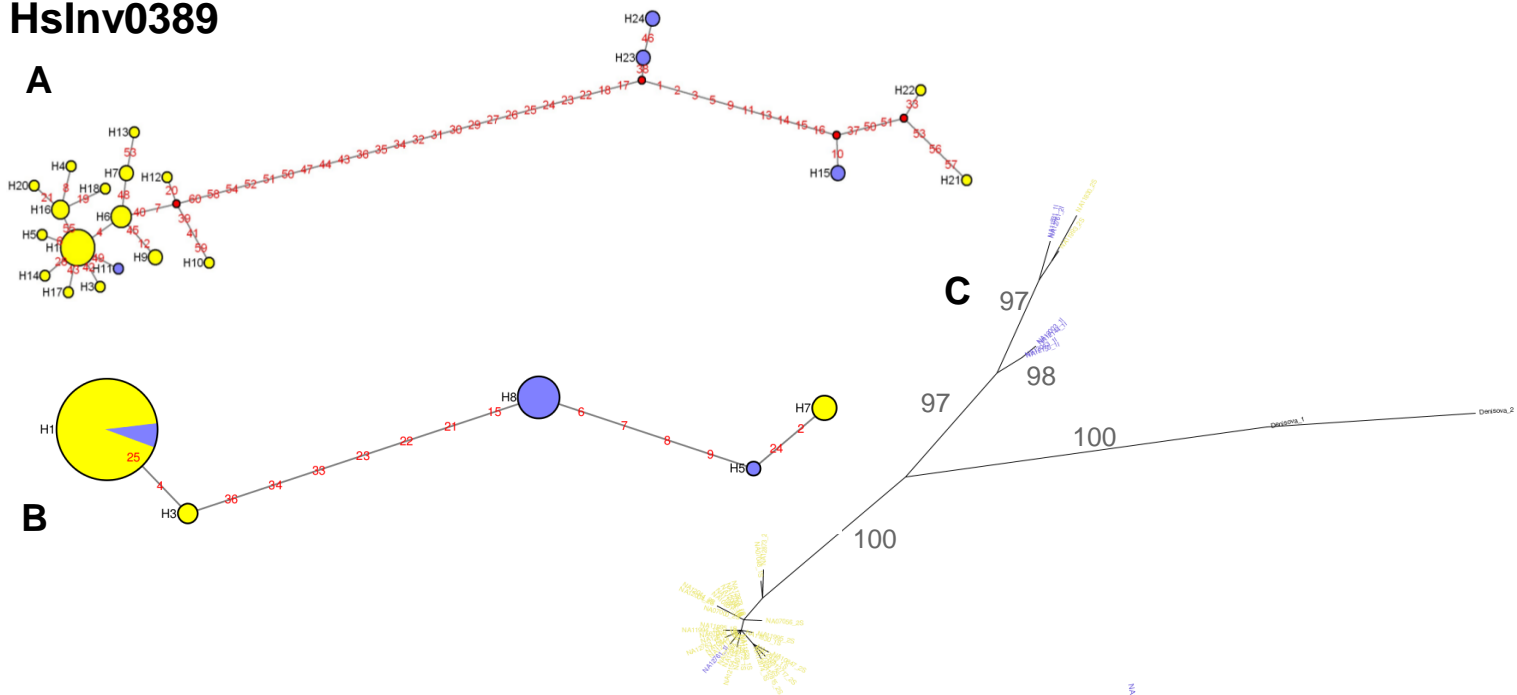

## HsInv0393

**A**

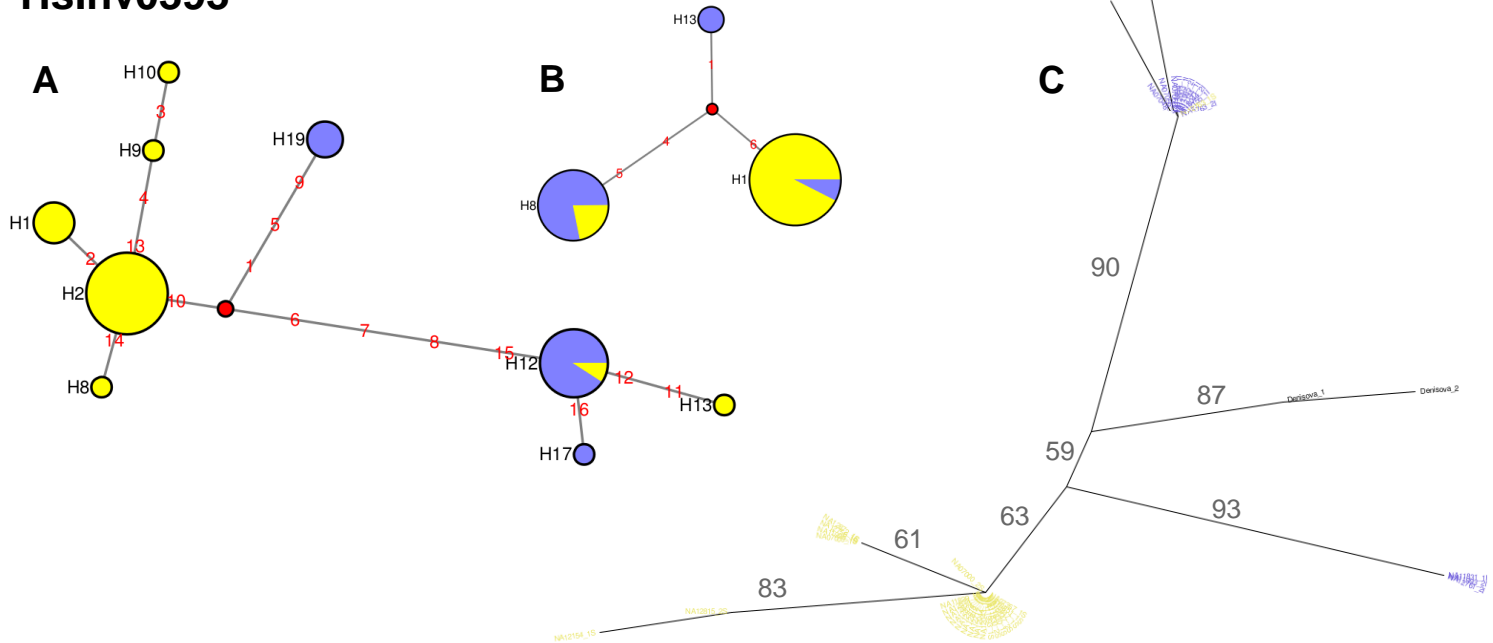

**HsInv0396**

**A**

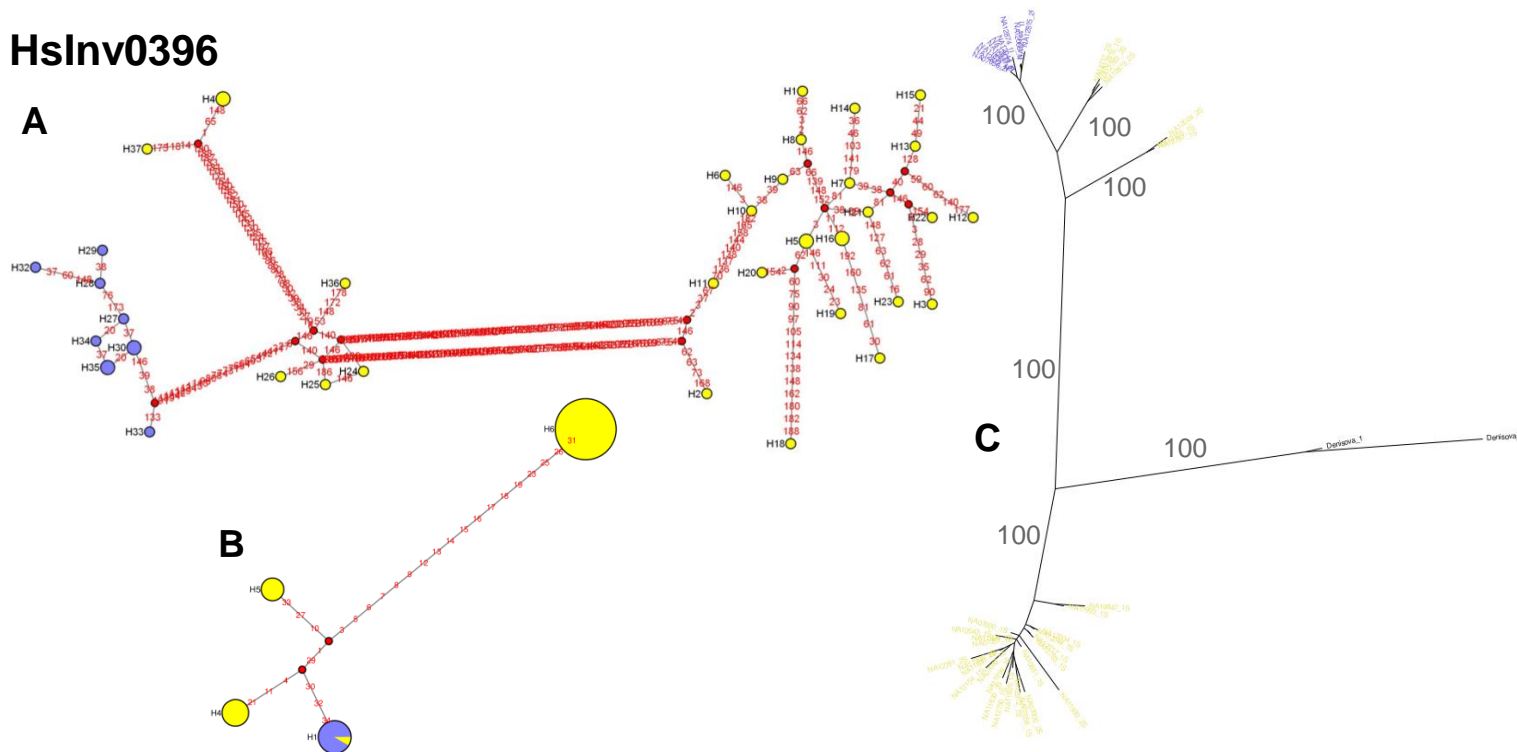

## HsInv0397

# A

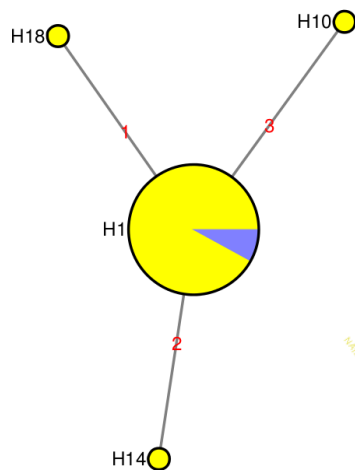

**C**

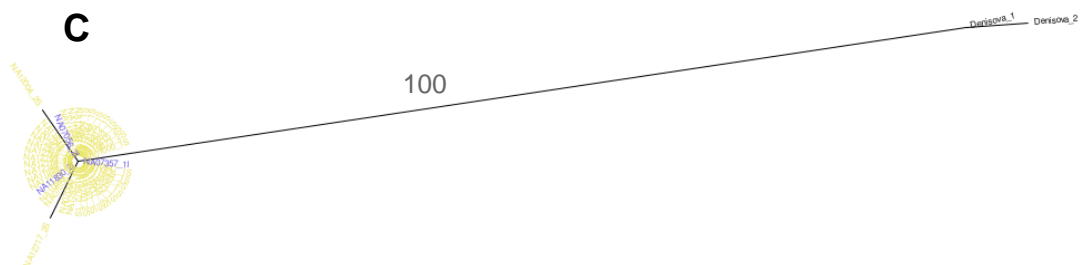

**HsInv0403**

**A**

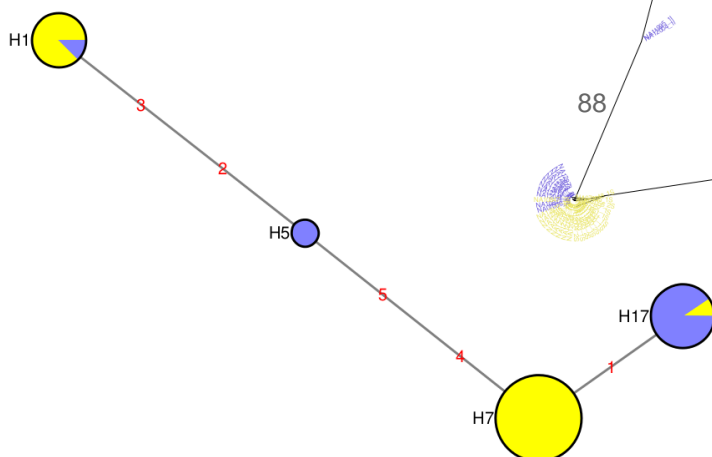

**C**

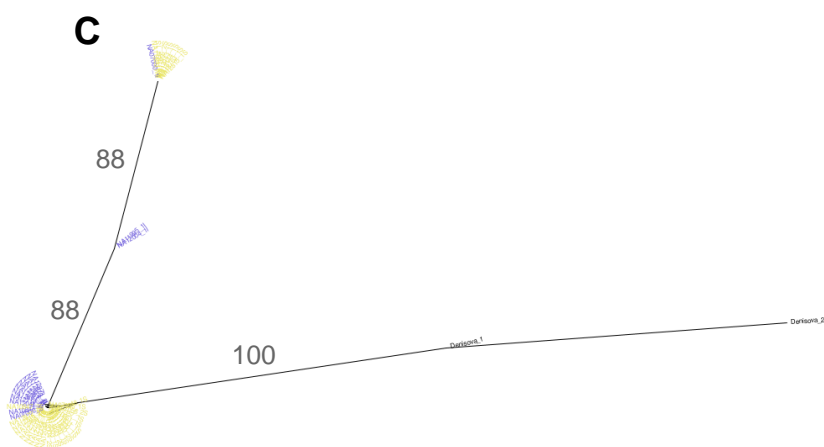

Supplement: Figure S2 — Median-Joining networks and Neighbor-Joining trees in CEU individuals for the 14 polymorphic inversions using the 1000 Genomes Project (A and C) and HapMap (B) SNP data. Haplotypes having the standard or inverted orientation are indicated in yellow and blue, respectively. In networks (A and B), circles represent the different haplotypes found for the region of the inversion and circle sizes are proportional to the frequency of each haplotype. Nucleotide changes between haplotypes are indicated as red numbers and red nodes correspond to hypothetical haplotypes. In trees (C), all sequences analyzed are represented in a different branch and black labels indicate the Denisovan sequences used as an outgroup. Bootstrap values >50 are shown based on 100 replications. Note that the two Denisovan sequences are not phased and do not represent real haplotypes. Alignments with chimpanzee (Pan troglodytes) and rhesus macaque (Macaca mulatta) have similar tree topology and location of the outgroup branch, although for many inversions the length of the sequence compared is much smaller. Trees were depicted with FigTree (http://tree.bio.ed.ac.uk/software/figtree/). (PDF) [file pgen.1004208.s002.pdf]

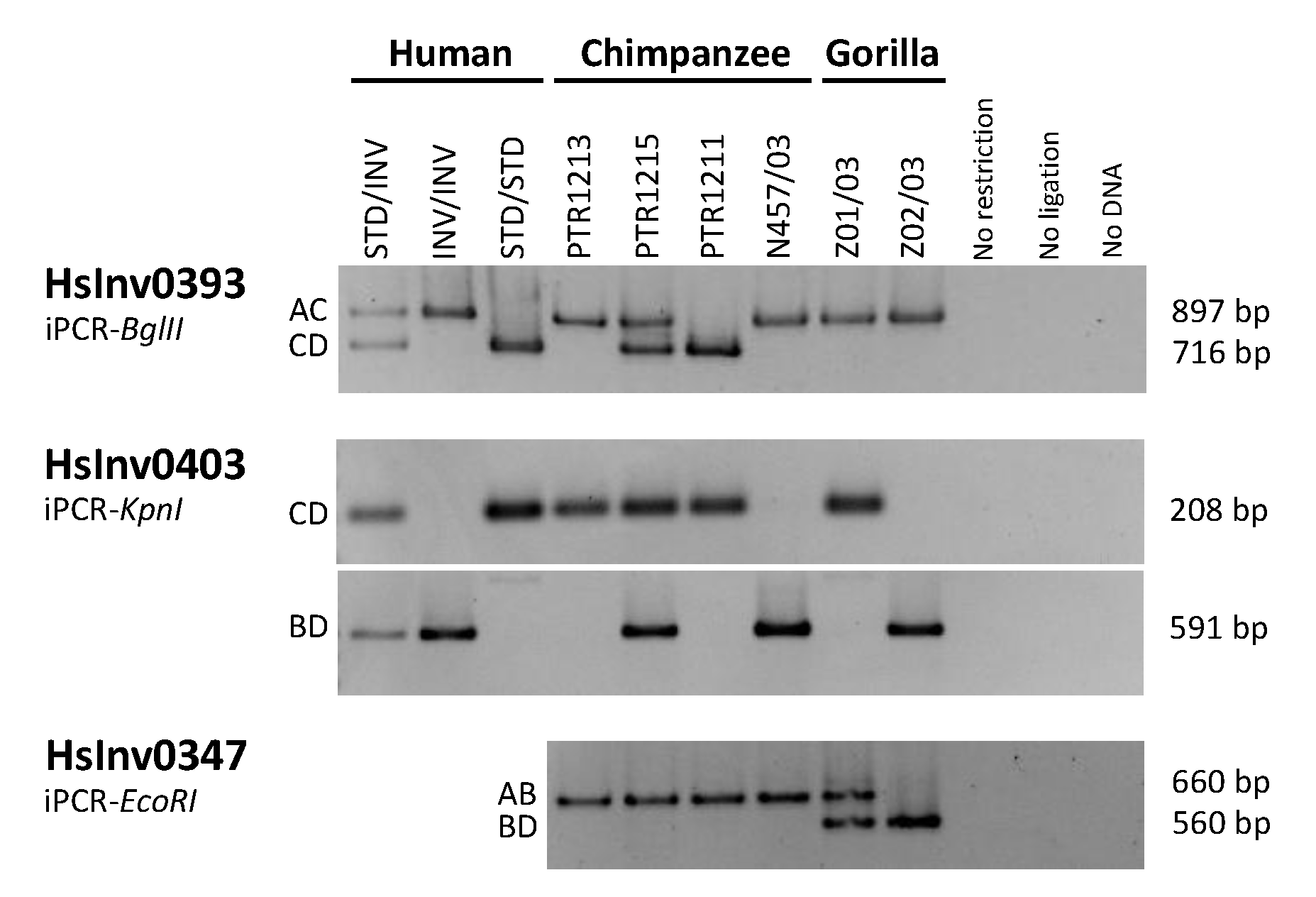

Supplement: Figure S3 — iPCR results for three human polymorphic inversions in non-human primate species. HsInv0393 shows both orientations in chimpanzees (CD and AC), but gorillas are inverted (AC). HsInv0403 shows both orientations (CD and BD) and is polymorphic in humans, chimpanzees and gorillas. HsInv0347 is polymorphic in gorillas (AB and BD), but the four chimpanzees are Std homozygotes (AB). Human samples are not included in HsInv0347 iPCR because a primer in region A specific for non-human primates was used. (TIF) [file pgen.1004208.s003.tif]
